# Supplementary material for: Assessment of undergraduate student knowledge, attitude, and practices towards COVID-19 in Debre Berhan University, Ethiopia
Source: PLoS One. 2021 May 18;16(5):e0250444. doi: 10.1371/journal.pone.0250444 (PMC8130923; doi:10.1371/journal.pone.0250444)
Supplement: S1 File — (DOCX) [file pone.0250444.s001.docx]

**A table of relevant demographic details**

| Sr o | Collage | Departments | Total under graduated student in each collage | Included collage data collection(randomly selected) |
| --- | --- | --- | --- | --- |
| 1 | **School of Computing Science** | - Information technology - Information system - Computer science - Software Engineering | **1231** |  |
| 2 | **College of Engineering** | - Electrical  and Computer Engineering - Mechanical engineering - Civil engineering - Chemical engineering - Construction technology and management - Industrial Engineering - Survey Engineering - Food Processing  Engineering | 4286 |  |
| 3 | **College of Health Science** | - Nursing - Midwifery - Health officer - Pediatrics Nursing - Neonatal Nursing - Surgical Nursing - Medical Laboratory Science | 616 |  |
| 4 | **College of** **Medicine** | - Anesthesia - Medicine - Pharmacy | 263 |  |
| 5 | **College of Natural and Computational Science** | - Biology - Chemistry - Physics - Mathematics - Sport science - Statistics - Biotechnology - Geology | 1231 |  |
| 6 | **College of Business and Economics** | - Management - Economics - Accounting and Finance - Tourism Management - Logistics and Supply Chain Management - Marketing Management | 1257 |  |
| 7 | **College of Agriculture and Natural Resource Science** | - Plant science - Animal science - Natural resource management - Water resource and irrigation management - Horticulture - Agricultural Economics | 741 |  |
| 8 | **College of Social Science and Humanities** | - Geography and Environmental Studies - History and heritage management - Sociology - Psychology - English language and literature - Amharic - Civics and ethical education - Journalism and Communication | 1210 |  |
| 9 | **College of Law** | Law | 221 |  |
| 10 | College of Education | - Technical Drawing  /Summer/ - Business Education /Summer/ - Special Need Education / Summer/ | 1235 |  |
