# Supplementary material for: Assessment of undergraduate student knowledge, attitude, and practices towards COVID-19 in Debre Berhan University, Ethiopia
Source: PLoS One. 2021 May 18;16(5):e0250444. doi: 10.1371/journal.pone.0250444 (PMC8130923; doi:10.1371/journal.pone.0250444)
Supplement: S2 File — (DOCX) [file pone.0250444.s002.docx]

**Questionnaire of knowledge, attitudes, and practice towards COVID-19**

Knowledge, attitude, and skills interview about **COVID-19**

This questionnaire was developed in 2012 to determine the level of knowledge, attitudes and skills of students at Debre Berhan University. My name is _______________. I am a lecturer in the School of Nursing at Debre Berhan University and I am the Coordinator of Corona virus Research. The main purpose of this research is to determine the level of knowledge and understanding of students at Debre Berhan University about covad-19 disease and to identify the causes of this disease. This situation needs to be confirmed by research. The results of this study will help to generate ideas to help prevent the disease. In this study, you are expected to share your thoughts. It will not take you more than 20 minutes. Your comment is confidential and it is not known who gave it. Only members of the research team have a chance to see it. The study does not harm you. You can terminate the study at any time.

**Written conset form**

The purpose of the above research is to determine the level of knowledge, attitudes and skills among students of Debre Berhan University about covad-19 disease and to identify the causes of the disease; I was told that my decision was based on my willingness to participate in the study, that my comments were confidential, and that I would not be harmed.

Respondent's signature ________________ date _____________________

Questionnaire Completion Date: ________ Time: _______ End Date: _________.

Data Collector Name ________________________ Signature _________

Instructions: Please circle the respondent's answers in front of the questions or write the respondent's words in the space on the right.

**Socio-demographic Characteristics of DBU Students**

1. Sex A. female B. male
2. Age in years--------------
3. Marital status

A. single B. married C. divorced

1. Religion

A. orthodox Christian B. Muslim C. protestant D. others

1. Residence

A. rural B. urban

1. Year of study

A. First B. second C. third d. fourth and fifth

1. Monthly pocket money-- -------------
2. Source of information about Novel coronavirus
3. News media(TV, magazines, newspapers, and radio) B. social media (Facebook, Twitter,Whatsapp, YouTube, Instagram, telegram)

C. official government websites

D. friends and family

**Knowledge** (correct rate, % of the total sample)

1. The main clinical symptoms of COVID-19 are fever, fatigue, dry cough, and shortness of breath.
2. True B. false C.I don’t know
3. Unlike the common cold, stuffy nose, runny nose, and sneezing are less common in persons infected with the COVID-19 virus.

A. True B. false C. I don’t know

1. There currently is no effective cure for COVID-2019, but early symptomatic and supportive treatment can help most patients recover from the infection.

A. True, B. false C. I don’t know

1. Not all persons with COVID-2019 will develop to severe cases. Only those who are elderly, have chronic illnesses, and are obese are more likely to be severe cases.

A. True, B. false C. I don’t know

1. COVID-19 is transmitted through air, contact, fecal-oral routes

A. True, B. false C. I don’t know

1. Eating or contacting wild animals would result in the infection by the COVID-19 virus.

A. True, B. False C. I don’t know

1. Persons with COVID-2019 cannot infect the virus to others when a fever is not present.

A. True B. false C. I don’t know

1. The COVID-19 virus spreads via respiratory droplets of infected individuals.

A. True B. false C. I don’t know

1. Ordinary residents can wear general medical masks to prevent the infection by the COVID-19 virus.

A. True, B. false C. I don’t know

1. It is not necessary for children and young adults to take measures to prevent the infection by the COVID-19 virus.

A. True, B. false C. I don’t know

1. To prevent the infection by COVID-19, individuals should avoid going to crowded places such as train stations and avoid taking public transportations.

A. True, B. false C. I don’t know

1. Isolation and treatment of people who are infected with the COVID-19 virus are effective ways to reduce the spread of the virus.

A. True B. false C. I don’t know

1. People who have contact with someone infected with the COVID-19 virus should be immediately isolated in a proper place. In general, the observation period is 14 days.

A.True B.false C. I don’t know

**Attitudes**

1. Do you agree that COVID-19 will finally be successfully controlled?

A. Agree, B. disagree, C. I don’t know

1. I have no concern of being infected with COVID-19

A. Agree, B. disagree, C. I don’t know

1. Do you agree that washing hands with soap and water could help to prevent covid 19 virus transmission.
2. A. Agree, B. disagree, C. I don’t know
3. Do you have confidence that Ethiopia can win the battle against the COVID-19 virus?

A. Agree, B. disagree, C. I don’t know

**Practices**

1. In recent days, have you gone to any crowded place?

A. Yes, B. no

1. In recent days, have you worn a mask when leaving home?
2. Yes, B. no
3. Do you wash your hands after sneezing or coughing
4. Yes B. no
5. **Do you touch your face, nose, or mouth with your unclean hands?**
6. frequently B. sometime
7. Do you cover your mouth and nose with an elbow or tissue while coughing, sneezing

A. Yes B. no

1. In recent days, have you maintain social distance at least 1 metre (3 feet) between yourself and anyone who is coughing or sneezing
2. Yes B. NO

**ስለ ኮረና ባይረስ እዉቀት፣አመለካከት ና ክህሎት ቃለመጠይቅ**

ይህ መጠይቅ የተዘጋጀዉ በ2012 ዓ.ም በደብረብርሃን ዮኒቨርስቲ ተማሪዎች ስለ ኮሮና ነቫይረስ ያላቸዉን እዉቀትና አመለካካት ና ክህሎት ደረጃ ለማወቅ እንዲሁም የዚህን በሽታ መንስኤዎችን ለመለየት ነዉ፡፡

ስሜ_____________________ ይባላል፡፡ በ ደብረ ብርሃን ዩኒቨርሲቲ የነርሲንግ ት/ት ከፍል መምህር ሲሆን የኮሮና ቫይረስ ጥናት አስተባባሪ ነኝ ፡፡ይህ የምርምር ዋና አላማዉ በደብረብርሃን ዩኒቨርስቲ ውስጥ ያሉ ተማሪዎች ስለ ኮቨድ-19 በሽታ ያላቸዉን እዉቀትና ግንዛቤ ደረጃ ለማወቅ እንዲሁም የዚህን በሽታ መንስኤዎችን ለመለየት ነዉ፡፡ ተማሪዎች ስለ ኮቨድ በሽታ ያላቸዉ የእዉቀት እና ግንዛቤ ደረጃ የተለያየ ሊሆን ይችላል፡፡ይህ ሁኔታ ደግሞ በጥናት መረጋገጥ ይፈልጋል፡፡ የዚህ ጥናት ዉጤት የኮቨድ በሽታን ለመከላከል የሚያግዙ ሃሳቦችን ለማመንጨት ያግዛል፡፡ በዚህ ጥናት ከአንተ/ች የሚጠበቀዉ ያለህን/ሽን ሃሳብ ማካፈል ነዉ፡፡ከ 20 ደቂቃ በላይ አይወስድብህም፡፡ የሰጠኸዉ/ሽዉ ሃሳብ ሚስጢራዊነቱ የተጠበቀ እና ማን ሃሳብ እንደሰጠ የማይታወቅ ነዉ፡፡የተሰጠዉን ሃሳብ የማየት እድል ያላቸዉ የምርምሩ ቡድን አባላት ብቻ ናቸዉ፡፡ጥናቱ በአንተ/አንቺ ላይ ምንም ዓይነት ጉዳት አያመጣም፡፡ ጥናቱን በማንኛዉም ሰዓት ማቋረጥ ትችላለህ፡፡

**የስምምነት ዉል**

ከላይ የተጠቀሰዉ የምርምር አላማ በደብረብርሃን ዩኒቨርስቲ ተማርዎች መካከል ስለ ኮቨድ -19 በሽታ ያላቸዉን እዉቀት፣አመለካከት ና ክህሎት ደረጃ ለማወቅ እንዲሁም የዚህን በሽታ መንስኤዎችን ለመለየት መሆኑ፤ በጥናቱ ለመሳተፍ በሙሉ ፈቃደኝነት ላይ የተመሰረተ መሆኑን፣የሰጠሁት ሃሳብ ሚስጥራዊነቱ የተጠበቀ፣በእኔ ላይ ምንም ዓይነት ጉዳት እደማይደርስብኝ ተነግሮኝና ተረድቼ በጥናቱ ተሳትፊያለሁ፡፡

የመልስ ሰጪዉ ፊርማ________________ቀን _____________________

መጠይቁ የሞላበት ቀን : ________ሰዓት: _______ያለቀበት ሰዓት: _________.

የመረጃ ሰብሳቢው ስም ________________________ፊርማ _________

ትዕዛዝ: እባክዎ መላሹ የሚሰጠዉን መልስ ከጥያቄዎች ፊት ለፊት ካሉት አማራጮቹ መካከል ያክብቡ ወይም መላሹ የሚገልፀዉን ሀሳብ በቀኝ በኩል ባለዉ ክፍት ቦታ ላይ ይፃፉ፡፡

**በመጀመሪያ ደረጃ የተማሪዎች የማህበራዊ እና ኢኮኖሚያዊ መረጃ መጠይቅ**

1. ጾታ ሀ. ወንድ ለ. ሴት

2. እድሜ ----------

3. የጋብቻ ሁኔታ ሀ. ያላግባ(ች) ለ. ያገባ(ች) ሐ. የፈታች(ታ)

4. ሐይማኖት ሀ. ኦርቶዶክስ ክርስትያን ለ. ሙስሊም ሐ. ፕሮቴስታንት መ. ሌላ ካለ ይግለጹ

5. የትዉልድ ቦታ ሀ. ከተማ ለ. ገጠር

6. ስንተኛ አመት ተማሪ ነህ(ሽ) -----

7. የወር ገቢ መጠን ስንት ነው-----------

8. ስለ ኮሮና ባይረስ የመረጃ ምንጭ ምንድ ነዉ

ሀ. የመንግስት ሚዲያ ለ. ሶሻል ሚዲያ ሐ. የመንግስት የመረጃ መረብ መ. ከጓደኛ ና ቤተሰብ

**እውቀትን የሚመለከቱ ጥያቅዎች**

1. ከፍተኛ ትኩሳት፣ ራስምታት፣ ደረቅሳል ና አጠቃላይ የሰዉነት ህመም የኮቢድ -19 ባይረስ ዋና የህመሙ ምልክት ናቸው

ሀ. እውነት ለ. ሀሰት ሐ. አላዉቀዉም

2. ምንም እንኮን እስከዚህ ሰዓት ኮቢድ-19 ባይረስን የሚፈዉስ መድሐኒት ባይኖርም ፣ነገር ግን ቅድመ ምርመራ ና ክብካቢ በማድረግ ብዙ በሽተኞችን እንዲያገግሙ ይረዳቸዋል.

ሀ. እውነት ለ. ሀሰት ሐ. አላዉቀዉም

3. ኮቢድ-19 ሁሉንም ሰው የከፋ በሸታ ደረጃ ላይ አይደርስም. ነገር ግን አዛውንቶችና ሌላ ተጓዳኝ በሽታ ያለባቸውን ሰዎችን በተለየ መልኩ የከፋ ደረጃ ሊያደርስባቸው ይችላል፡፡

ሀ. እውነት ለ. ሀሰት ሐ. አላዉቀዉም

4. ኮቢድ-19 በንክኪ፣ በትንፋሽ ና ከእጅ ወደ አፍ ይተላለፋል፡፡

ሀ. እውነት ለ. ሀሰት ሐ. አላዉቀዉም

5. የዱር እንስሳትን መመገብም ሆነ ንክኪ ማድረግ ኮቢድ -19 በሽታ ያስተላልፋል፡፡

ሀ. እውነት ለ. ሀሰት ሐ. አላዉቀዉም

6. አንድ ሰው ከፍተኛ ሙቀት ከሌለው ኮቢድ-19 በሽታን ወደ ሌላ ስዉ አያሰተላልፍም

ሀ. እውነት ለ. ሀሰት ሐ. አላዉቀዉም

7. የኮቢድ -19 በሽታን ስርጭት ለመቀነስ ማንኛውም ሰው የፊት ጭንብል ማድረግ አለበት

ሀ. እውነት ለ. ሀሰት ሐ. አላዉቀዉም

8. ህጸናትና ወጣቶች የኮቢድ-19 በሽታን ለመከላከል ምንም አይነት ጥንቃቄ ማድረግ አይጠበቅባቸዉም

ሀ. እውነት ለ. ሀሰት ሐ. አላዉቀዉም

9. የኮቢድ-19 በሸታን ስርጭት ለመቀነስ ግለሰቦች ወደ ተጨናነቀ ቦታ ለምሳሌ ወደ ባቡር ጣቢያ እና የህዝብ ትራንስፖት መሂድ የለባቸዉም

ሀ. እውነት ለ. ሀሰት ሐ. አላዉቀዉም

10. በኮቢድ-19 የተያዙ ሰዎችን በመነጠል እና አስፍላጊዉን እንክብካቢ በማድረግ የበሽታዉን ስርጭት መቀነስ ይቻላል

ሀ. እውነት ለ. ሀሰት ሐ. አላዉቀዉም

11. እጅ መታጠብ በሚያስሉበት ጊዚ አፍና አፍጫን መሸፍን እና ከበሽተኛ ሰዉ ጋር ንክኪ አለማድረግ የበሽታዉ ስርጭት ይቀንሳል

ሀ. እውነት ለ. ሀሰት ሐ. አላዉቀዉም

**አመለካከትን የሚመለከቱ ጥያቅዎች**

1.የኮቢድ-19 ስርጭት ልንቆጣጠረ እንእላለን ብለው ይስማማሉ

ሀ. እስማማለሁ ለ. ገለልተኛ ሐ. አልስማማም

2. በኮቢድ-19 እያዛለሁ የሚል እሳቢ አሎት

ሀ. እስማማለሁ ለ. ገለልተኛ ሐ. አልስማማም

3. እጅን በሳሙና እና በዉሀ መታጠብ የኮርናን በሸታ ይከላከላል ብለው ያስባሉ

ሀ. እስማማለሁ ለ. ገለልተኛ ሐ. አልስማማም

4. ኢትዮጵያ የኮቢድ-19 በሽታን ስርጭት ልትቆጣጠር እደምችል ሙሉ እምነት አሎት

ሀ. እስማማለሁ ለ. ገለልተኛ ሐ. አልስማማም

5. ኮቢድ-19 ገዳይ በሽታ ነዉ ብለዉ ያስባሉ

ሀ. እስማማለሁ ለ. ገለልተኛ ሐ. አልስማማም

**ክህሎትን የሚመለከቱ ጥያቄዎች**

1. በቅርብ ቀን ወደ ተጨናነቀ ቦታ ሂደው ነበር

ሀ. አዎ ለ. አልሄድኩም

2. በቅርብ ቀን ከቤትዎ ሲወጡ የፊት ጭንብል አጥልቀው ነበር

ሀ. አዎ ለ. አላጠለኩም

3. በቅርቡ እጅዎን በንጹህ ዉሀና ሳሙና፣ እንዲሁም በአልኮል ታጥበዋል

ሀ. አዎ ለ. አልታጠብኩም

4. በእርስዎና ሌላ በሚያስል ወይም በሚያስነጥስ ሰው መካከል ቢያንስ 1 ሜትር/ 3 ጫማ/ ልዩነት እንዲኖር ያደርጋሉ

ሀ. አዎ ለ. አላደርግም

5. ባልታጠበ እጅዎ ፊትን ፣አይንን፣አፍንጫን ና አፍዎን ከመንካት ተቆጥበዋል

ሀ. አዎ ለ. አልተቆጠብኩም

6. በሚያስሉ ወይም በሚያስነጥሱ ግዜ አፍ ና አፍንጫቆን በእጅ ክርንዎ ወይም በሶፍት ይሸፍናሉ

ሀ. አዎ ለ. አልሸፍንም
