# Supplementary material for: Assessment of undergraduate student knowledge, attitude, and practices towards COVID-19 in Debre Berhan University, Ethiopia
Source: PLoS One. 2021 May 18;16(5):e0250444. doi: 10.1371/journal.pone.0250444 (PMC8130923; doi:10.1371/journal.pone.0250444)
Supplement: Attachment — Submitted filename: PONE-D-20-14158_R4.pdf [file pone.0250444.s009.pdf]

PLOS ONE

Assessment of undergraduate student knowledge, attitude, and practices, towards COVID-19 in Debre Berhan University, Ethiopia

--Manuscript Draft--

**Manuscript Number:**	PONE-D-20-14158R4
**Article Type:**	Research Article
**Full Title:**	Assessment of undergraduate student knowledge, attitude, and practices, towards COVID-19 in Debre Berhan University, Ethiopia
**Short Title:**	Assessment of undergraduate student knowledge, attitude, and practices, towards COVID-19
**Corresponding Author:**	Yared Asmare Aynalem<br>Debre Berhan University<br>Debre Berhan, ETHIOPIAN ETHIOPIA
**Keywords:**	knowledge; Attitude; Practice; COVID-19; Ethiopia
**Abstract:**	Abstract<br>Background: Novel coronavirus-2019 (COVID-19) is a highly infectious disease that has caused a global pandemic. As of July 2020, there were 8,475 confirmed cases of COVID-19 in Ethiopia, and a total of 52 cases and 1 death were reported in Debre Berhan where this study was conducted. Under these conditions, we sought to assess what undergraduate students at Debre Berhan University knew about COVID-19 and how it shaped their attitudes and practices regarding this disease.<br>Objective: The aim of the current study was to assess undergraduate student knowledge, attitudes and practices towards COVID-19 in Debre Berhan University, Ethiopia.<br>Methods : A cross-sectional survey was conducted from March 18–24, 2020 among undergraduate students at Debre Berhan University. A two-stage cluster sampling technique was employed with a total sample size of 634. Proportional allocation of samples was used to the randomly selected colleges, and a systematic random sampling technique was employed to recruit the students. The data were checked for completeness, coded, entered into Epi Data VS 3.1, and then exported into STATA™ Version 14 software for analysis. Descriptive statistics were conducted. Binary logistic regression analyses were used to identify factors. Factors were selected with the entry method. Adjusted odds ratios (AORs) and their 95% confidence intervals (CIs) were used to assess the associations between variables and knowledge, attitude and practices (KAP).<br>Results: From a total of 546 included participants, more than half of them, 307 (57%) were males. Seventy-three percent of them heard about novel coronavirus from social media. In this study, 73.8% of the participants were knowledgeable, and their overall attitude was favorable. Approximately 71.4% correctly responded that the main clinical symptoms of COVID-19 are fever, fatigue, dry cough, and shortness of breath. Nearly half, 229 (42%) of the students agreed that they had no concern of being infected with COVID-19. Moreover, most participants showed poor practices; more than half of the study subjects were not maintaining a physical distance. In multivariable analyses, people 25 and older (AOR = 1.6, 95% CI; 1.2, 4.6) and those who lived in urban areas (AOR = 4.3, 95% CI; 2.6, 15.8) were significantly associated with being knowledgeable about COVID-19. Furthermore, we found that the source of information about COVID-19 (AOR = 2.3, 95% CI; 1.6, 8.7) was significantly associated with the attitude undergraduate students had about COVID-19.<br>Conclusion: The undergraduate students at Debre Berhan University were moderately knowledgeable about COVID-19 and had an optimistic attitude towards its resolution. However, this optimism may lead to poor public health practices within this community. Therefore, greater efforts need to be made through more comprehensive and directed actions and awareness campaigns to increase the knowledge, attitude and practice of the students.
**Order of Authors:**	Yared Asmare Aynalem
	Tadess Yirga Akalu

Powered by Editorial Manager® and ProduXion Manager® from Aries Systems Corporation

	Birhan Gebresellassie
	Nigussie Tadesse Sharew
	Wondimeneh Shibabaw Shiferaw
	Hilina Ketema Assefa
**Opposed Reviewers:**	
**Response to Reviewers:**	Dear Academic Editor!<br>PLOSE ONE<br>Response to Academic Editor<br>I am pleased to resubmit revision (R4) for publication version of "Assessment of undergraduate student knowledge, practices, and attitude towards COVID-19 in Debre Berhan University, Ethiopia for a review as original research in Plose one .The comments of the editor were highly insightful and enabled us to greatly improve the quality of our manuscript. Therefore, based on the editor's concerns we have made extensive edition in our manuscript. Especially we have extensively edited the manuscript again for language usage, spelling, and grammar before submitting the revised version. The formatting of the text and document (text sizes and grammatical errors) were also edited. In the following pages, we have addressed yours' concerns in a point by point format.<br>We look forward to hearing from you at your earliest convenience.<br>Thank you for your consideration of this manuscript!<br>Kind regards,<br><br>Yared Asmare Aynalem.<br>On behalf of authors<br><br><br>PONE-D-20-14158R3<br>Assessment of undergraduate student knowledge, attitude, and practices, towards COVID-19 in Debre Berhan University, Ethiopia<br>PLOS ONE<br><br>Dear Dr. Aynalem,<br>Thank you for submitting your manuscript to PLOS ONE. After careful consideration, we feel that it has merit but does not fully meet PLOS ONE's publication criteria as it currently stands. Therefore, we invite you to submit a revised version of the manuscript that addresses the points raised during the review process.<br>Response: Thank you very much for allowing us to revise our manuscript again. We have tried to response each concerns as much as possible. We have submitted a revised version of the manuscript<br>I thank you for the revisions that you have conducted, but I would urge you once more to carefully review the manuscript in terms of clarity and grammar. This is not the usual request for authors to ensure that the English language in the manuscript is adequate, but to ensure that the manuscript is readable and understandable. The paper has had several rounds of reviews and opportunities for these issues to be resolved, so I would be grateful if you could ensure they are resolved. It is disconcerting to find so many issues despite assurances that they have been resolved.<br>Response: Thank you for allowing us to edit it again. The comments of the editor and the reviewers were highly insightful and enabled us to greatly improve the quality of our manuscript. Therefore, based on your concerns we have made extensive edition in our manuscript. Your comments were highly insightful and enabled us to greatly improve the quality of our manuscript. Therefore, based on the editor's concerns we have made extensive edition in our manuscript. Especially we have extensively edited the manuscript again for language clarity thoroughly edited the manuscript for language usage, spelling, and grammar before submitting the revised version. The formatting of the text and document (text sizes and grammatical errors) were also edited.<br><br>In addition to the comments and recommendations mentioned by the reviewers, I have read the manuscript again and I would like the authors to resolve these pending matters:<br>Response: Dear our respected editor thank you very much for raising the following issues

Background

2nd para, why is the mortality compared with the flu?
Response: Dear our respected editor thanks you very much. The reason for comparing COVID-19 with the flu is that to show the proportion of mortality as a new outbreak. At the time of seasonal flue outbreak the mortality rate were almost 1%.more over covid mortality rate is almost more than 7 percent .This death rate is particularly alarming. If you are still not comfortable with this sentence, we will remove it.
End of 2nd para, and start of 3rd para, the data does not match- please review and clarify.
Response: Thanks you very much our respected editor .We have rewrite it again; please see the color change from the main document
Ethics statement
Review wording- "ethical approval commute" should be committee - please review across the manuscript. Review the paragraph for clarity.
Response: Thanks you very much our respected editor. We have copy edited it. see also the color change from the ma document
Response: Thank you dear editor. We have clearly descried the contribution on the on the online author contribution form.
Background
2nd para, says "2215 countries", must be a typo.
Response: thanks our beloved editor We have rewritten it. Please See the color change
Reference in pg 16 [9,10] appears in cursive font, please edit.
Response: Thanks .We has corrected it. Please see the color change
End of page: "study conducted in different parts of their world" - say "international" or say which parts of the world.
Response: Thanks .We tried to rephrase & replace with your suggestive appropriate word so called "international"
Methods
Please remove "Abve sea level and temp" unless relevant to the study. Please edit "Collage of ..." To "College of..."
Response: Thanks .We has corrected it. See the color change
Justify socio-demographic variables, marital status, religion - why collected?
Response: we would like to thanks our editor for raising these important concerns. The rational for collecting socio-demographic variables, marital status, and religion is as follows
1.In our country context, the distribution of COVID_19 is high among aged individual & most of adult generation perceived that they are less risky of contracting with it. For that they didn’t want to get even information on it. Therefore, we have collected it as a factor.
2.Most of the community including students talk as COVID is a sin of us.it is from alah,God. So that, not practicing a percussion. This document and risk assessment tool provides practical guidance and recommendations to support the special role of religious leaders, faith-based organizations, and faith communities in COVID-19 education, preparedness, and response.
3.Additionally, differ study conducted globally, shows that socio-demographic variables, marital status, religion are linked with COVID-19 KAP. Here, we examine how KAP varies across fundamental socio-demographic characteristics, including age, sex, civil status, individual disposable income, religion of. We have collected it to for identifying the gap & to intervene on it .
Was the data collection validated?
Was tool validated in students or only faculty?
"Ensure genuine replies..." - how did they do that?
Thanks dear reviewer..!!! The proposed tool was developed and validated by a multidisciplinary working group of infectious disease physicians, lecturers (Public health, nurse & environmental health professionals, and infectious diseases Public health professionals. The working group regularly reviewed literature to select important characteristics and outcomes for inclusion. We also used a
The development phase was conducted at Debre birhan University health science collage and the pilot validation phase was conducted at the nursing department students. We have also done a pretest from 5 % of the student. The team members formed a working group that met via telephone or video conferences at least biweekly
Ethics consideration

Commute = committee,Paper says participation anonymous but later on it mentioned that collects personal details like phone? Please clarify
Response: Thanks .We has corrected it. See the color change

Results
What was the total number of participants approached, to give the 86.1% rate? 634
Response: Thanks dear editor.as we have stated in sample size determination, we have included approached to a total of 634 participate.
Discussion
End of 1st para: please add evidence that "Students get regular update through the medias"
Response: Dear editor thanks for raising the concern. We have given a possible justifications based o our experience of giving the student about the outbreak COVID-19. We have given to them a health education program for all faculty of the university before the school was closed. Possibly this may increase their knowledge
2nd and 3rd para need punctuation mark at the end.
Response : Dear editor thanks for raising the concern. We have tried to edit all parts of the paper.

Response: thank you for your critical review dear editor. We have corrected it .See also the color change
Please submit your revised manuscript by Jan 24 2021 11:59PM. If you will need more time than this to complete your revisions, please reply to this message or contact the journal office at plosone@plos.org. When you're ready to submit your revision, log on to https://www.editorialmanager.com/pone/ and select the 'Submissions Needing Revision' folder to locate your manuscript file.
Please include the following items when submitting your revised manuscript:
A rebuttal letter that responds to each point raised by the academic editor and reviewer(s). You should upload this letter as a separate file labeled 'Response to Reviewers'.
A marked-up copy of your manuscript that highlights changes made to the original version. You should upload this as a separate file labeled 'Revised Manuscript with Track Changes'.

An unmarked version of your revised paper without tracked changes. You should upload this as a separate file labeled 'Manuscript'.
Response: Thank you. We have included ‘Response to Reviewers', 'Revised Manuscript with Track Changes & Manuscript separately
If you would like to make changes to your financial disclosure, please include your updated statement in your cover letter. Guidelines for resubmitting your figure files are available below the reviewer comments at the Editorial manager (https://www.editorialmanager.com/pone/)
Response: Thank you. We dot like to change the financial disclosure
We look forward to receiving your revised manuscript.
Kind regards,

Enrique Castro-Sánchez
Academic Editor
PLOS ONE
Reviewers' comments:
Reviewer's Responses to Questions
Comments to the Author
1. If the authors have adequately addressed your comments raised in a previous round of review and you feel that this manuscript is now acceptable for publication, you may indicate that here to bypass the “Comments to the Author” section, enter your conflict of interest statement in the “Confidential to Editor” section, and submit your "Accept" recommendation.

Reviewer #3: All comments have been addressed
Response: dear reviewer, we thank you for spending your valuable time to improve this paper. We have a grate appreciation for helping us
2. Is the manuscript technically sound, and do the data support the conclusions?
The manuscript must describe a technically sound piece of scientific research with data

	that supports the conclusions. Experiments must have been conducted rigorously, with appropriate controls, replication, and sample sizes. The conclusions must be drawn appropriately based on the data presented.<br>Reviewer #3: Yes<br>Response: Thank you dear reviewer<br>3. Has the statistical analysis been performed appropriately and rigorously?<br>Reviewer #3: Yes<br>Response: Thank you dear reviewer<br>4. Have the authors made all data underlying the findings in their manuscript fully available?<br>The PLOS Data policy requires authors to make all data underlying the findings described in their manuscript fully available without restriction, with rare exception (please refer to the Data Availability Statement in the manuscript PDF file). The data should be provided as part of the manuscript or its supporting information, or deposited to a public repository. For example, in addition to summary statistics, the data points behind means, medians and variance measures should be available. If there are restrictions on publicly sharing data—e.g. participant privacy or use of data from a third party—those must be specified.<br>Reviewer #3: Yes<br>Response: Thank you dear reviewer<br>5. Is the manuscript presented in an intelligible fashion and written in standard English?<br>PLOS ONE does not copyedit accepted manuscripts, so the language in submitted articles must be clear, correct, and unambiguous. Any typographical or grammatical errors should be corrected at revision, so please note any specific errors here.<br>Reviewer #3: Yes<br>Response: Thank you dear reviewer<br>6. Review Comments to the Author<br>Please use the space provided to explain your answers to the questions above. You may also include additional comments for the author, including concerns about dual publication, research ethics, or publication ethics. (Please upload your review as an attachment if it exceeds 20,000 characters)<br>Reviewer #3: Authors have adequately addressed my comments in this new version of the article. Discussion section provides more concrete information.<br>Response: Thank you dear reviewer. It is your effort too<br>7. PLOS authors have the option to publish the peer review history of their article (what does this mean?). If published, this will include your full peer review and any attached files.<br>If you choose “no”, your identity will remain anonymous but your review may still be made public.<br>Do you want your identity to be public for this peer review? For information about this choice, including consent withdrawal, please see our Privacy Policy.<br>Reviewer #3: Yes: Ramon Flecha<br>Response: Thank you dear reviewer<br><br>Dear all (both the reviewer ad editors),we would like to appreciate all your effort for your effort to improve our paper .may God paid all your effort. We have get differ research skills from your point of view<br>Thanks
**Additional Information:**	
**Question**	**Response**
**Financial Disclosure**<br><br>Enter a financial disclosure statement that describes the sources of funding for the work included in this submission. Review the submission guidelines for detailed requirements. View published research articles from *PLOS ONE* for specific examples.	The author(s) received no specific funding for this work.

This statement is required for submission and **will appear in the published article** if the submission is accepted. Please make sure it is accurate.<br><br>**Unfunded studies**<br>Enter: *The author(s) received no specific funding for this work.*<br><br>**Funded studies**<br>Enter a statement with the following details:<br>• Initials of the authors who received each award<br>• Grant numbers awarded to each author<br>• The full name of each funder<br>• URL of each funder website<br>• Did the sponsors or funders play any role in the study design, data collection and analysis, decision to publish, or preparation of the manuscript?<br>• **NO** - Include this sentence at the end of your statement: *The funders had no role in study design, data collection and analysis, decision to publish, or preparation of the manuscript.*<br>• **YES** - Specify the role(s) played.<br><br>* typeset	
**Competing Interests**<br><br>Use the instructions below to enter a competing interest statement for this submission. On behalf of all authors, disclose any competing interests that could be perceived to bias this work—acknowledging all financial support and any other relevant financial or non-financial competing interests.<br><br>This statement **will appear in the published article** if the submission is accepted. Please make sure it is accurate. View published research articles from *PLOS ONE* for specific examples.	The authors have declared that no competing interests exist.

Powered by Editorial Manager® and ProduXion Manager® from Aries Systems Corporation

**NO authors have competing interests**<br><br>Enter: *The authors have declared that no competing interests exist.*<br><br>**Authors with competing interests**<br><br>Enter competing interest details beginning with this statement:<br><br>*I have read the journal's policy and the authors of this manuscript have the following competing interests: [insert competing interests here]*<br><br>* typeset	
**Ethics Statement**<br><br>Enter an ethics statement for this submission. This statement is required if the study involved:<br><br>• Human participants<br>• Human specimens or tissue<br>• Vertebrate animals or cephalopods<br>• Vertebrate embryos or tissues<br>• Field research<br><br>Write "N/A" if the submission does not require an ethics statement.<br><br>General guidance is provided below. Consult the submission guidelines for detailed instructions. **Make sure that all information entered here is included in the Methods section of the manuscript.**	Ethics consideration<br>Ethical clearance for this study was obtained from the Institute of Medicine and College of Health Sciences, DBU. It was approved by the University ethical approval commute with the approval number of Ref. No.DBU.R.D136/02/12. Participants also gave a written informed consent prior to data collection after the participates are informed that their participation is completely voluntary and anonymous, if they decide to take part, still free to withdraw at any time, all information that is collected will kept strictly confidential, personal details such as phone number and address will not be revealed to people outside the research teams. Confidentiality of the study participants' information was maintained throughout the study by making the participants' information anonymous.

**Format for specific study types**<br><br>**Human Subject Research (involving human participants and/or tissue)**<br>• Give the name of the institutional review board or ethics committee that approved the study<br>• Include the approval number and/or a statement indicating approval of this research<br>• Indicate the form of consent obtained (written/oral) or the reason that consent was not obtained (e.g. the data were analyzed anonymously)<br><br>**Animal Research (involving vertebrate animals, embryos or tissues)**<br>• Provide the name of the Institutional Animal Care and Use Committee (IACUC) or other relevant ethics board that reviewed the study protocol, and indicate whether they approved this research or granted a formal waiver of ethical approval<br>• Include an approval number if one was obtained<br>• If the study involved *non-human primates*, add *additional details* about animal welfare and steps taken to ameliorate suffering<br>• If anesthesia, euthanasia, or any kind of animal sacrifice is part of the study, include briefly which substances and/or methods were applied<br><br>**Field Research**<br><br>Include the following details if this study involves the collection of plant, animal, or other materials from a natural setting:<br>• Field permit number<br>• Name of the institution or relevant body that granted permission	
**Data Availability**<br><br>Authors are required to make all data underlying the findings described fully available, without restriction, and from the time of publication. PLOS allows rare exceptions to address legal and ethical concerns. See the PLOS Data Policy and FAQ for detailed information.	Yes - all data are fully available without restriction

A Data Availability Statement describing where the data can be found is required at submission. Your answers to this question constitute the Data Availability Statement and **will be published in the article**, if accepted.<br><br>**Important:** Stating 'data available on request from the author' is not sufficient. If your data are only available upon request, select 'No' for the first question and explain your exceptional situation in the text box.<br><br>Do the authors confirm that all data underlying the findings described in their manuscript are fully available without restriction?	
**Describe where the data may be found in full sentences. If you are copying our sample text, replace any instances of XXX with the appropriate details.**<br><br>• If the data are **held or will be held in a public repository**, include URLs, accession numbers or DOIs. If this information will only be available after acceptance, indicate this by ticking the box below. For example: *All XXX files are available from the XXX database (accession number(s) XXX, XXX.)*.<br>• If the data are all contained **within the manuscript and/or Supporting Information files**, enter the following: *All relevant data are within the manuscript and its Supporting Information files.*<br>• If neither of these applies but you are able to provide **details of access elsewhere**, with or without limitations, please do so. For example:<br><br>*Data cannot be shared publicly because of [XXX]. Data are available from the XXX Institutional Data Access / Ethics Committee (contact via XXX) for researchers who meet the criteria for access to confidential data.*<br><br>*The data underlying the results presented in the study are available from (include the name of the third party*	All materials are attached as supplementary materials, and all other information is included in the manuscript.

and contact information or URL).<br>• This text is appropriate if the data are owned by a third party and authors do not have permission to share the data.<br><br>* typeset	
Additional data availability information:	

Dear Academic Editor!

PLOSE ONE

Response to Academic Editor

I am pleased to resubmit revision (R4) for publication version of "**Assessment of undergraduate student knowledge, practices, and attitude towards COVID-19 in Debre Berhan University**, Ethiopia for a review as original research in **Plose one .**The comments of the editor were highly insightful and enabled us to greatly improve the quality of our manuscript. Therefore, based on the editor's concerns we have made extensive edition in our manuscript. Especially we have extensively edited the manuscript again for language usage, spelling, and grammar before submitting the revised version. The formatting of the text and document (text sizes and grammatical errors) were also edited. In the following pages, we have addressed yours' concerns in a point by point format.

We look forward to hearing from you at your earliest convenience.

Thank you for your consideration of this manuscript!

Kind regards,

Yared Asmare Aynalem.

On behalf of authors

Manuscript Click here to access/download;Manuscript;Revised Manuscript R4..docx

Assessment of undergraduate student knowledge, attitude, and practices, towards COVID-19 in Debre Berhan University, Ethiopia

Yared Asmare Aynalem[1*] Tadess Yirga Akalu[2], Birhan Gebresellassie[1], Nigussie Tadesse Sharew[1,] Hilina Ketema Assefa[1],Wondimeneh Shibabaw Shiferaw[1]

[1]Department of Nursing, College of Health Science, Debre Berhan University, Debre Berhan, Ethiopia

[2]Department of Nursing, College of Health Science, Debre Markos University, Debre Markos, Ethiopia

[*]Corresponding author: yaredasmare123@gmail.com

Abstract

Background: Novel coronavirus-2019 (COVID-19) is a highly infectious disease that has caused a global pandemic. As of July 2020, there were 8,475 confirmed cases of COVID-19 in Ethiopia, and a total of 52 cases and 1 death were reported in Debre Berhan where this study was conducted. Under these conditions, we sought to assess what undergraduate students at Debre Berhan University knew about COVID-19 and how it shaped their attitudes and practices regarding this disease.

Objective: The aim of the current study was to assess undergraduate student knowledge, attitudes and practices towards COVID-19 in Debre Berhan University, Ethiopia.

Methods: A cross-sectional survey was conducted from March 18–24, 2020 among undergraduate students at Debre Berhan University. A two-stage cluster sampling technique was employed with a total sample size of 634. Proportional allocation of samples was used to the randomly selected colleges, and a systematic random sampling technique was employed to recruit the students. The data were checked for completeness, coded, entered into Epi-Data VS 3.1, and then exported into STATA™ Version 14 software for analysis. Descriptive statistics were conducted. Binary logistic regression analyses were used to identify factors. Factors were selected with the entry method. Adjusted odds ratios (AORs) and their 95% confidence intervals (CIs) were used to assess the associations between variables and knowledge, attitude and practices (KAP).

Results: From a total of 546 included participants, more than half of them, 307 (57%) were males. Seventy-three percent of them heard about novel coronavirus from social media. In this study, 73.8% of the participants were knowledgeable, and their overall attitude was favorable. Approximately 71.4% correctly responded that the main clinical symptoms of COVID-19 are fever, fatigue, dry cough, and shortness of breath. Nearly half, 229 (42%) of the students agreed that they had no concern of being infected with COVID-19. Moreover, most participants showed poor practices; more than half of the study subjects were not maintaining a physical distance. In multivariable analyses, people 25 and older (AOR = 1.6, 95% CI; 1.2, 4.6) and those who lived in urban areas (AOR = 4.3, 95% CI; 2.6, 15.8) were significantly associated with being knowledgeable about COVID-19. Furthermore, we found that the source of information about

COVID-19 (AOR = 2.3, 95% CI; 1.6, 8.7) was significantly associated with the attitude undergraduate students had about COVID-19.

Conclusion: The undergraduate students at Debre Berhan University were moderately knowledgeable about COVID-19 and had an optimistic attitude towards its resolution. However, this optimism may lead to poor public health practices within this community. Therefore, greater efforts need to be made through more comprehensive and directed actions and awareness campaigns to increase the knowledge, attitude and practice of the students.

Background

The novel coronavirus-2019 (COVID-19) is a newly discovered infectious disease that can cause severe illness in humans[1]. It was first detected in December 2019 in Wuhan, China, and quickly spread around the world. Fever, dry cough, fatigue, myalgia, and dyspnea are considered the main clinical symptoms of this disease. In China, 18.5% of the patients with COVID-19 developed acute respiratory distress syndrome, septic shock, metabolic acidosis, and bleeding. In more severe cases, it can cause pneumonia, severe acute respiratory syndrome, kidney failure, and even death[2, 3].

As of July 17, 2020, there were 13,961,427 confirmed cases and 592,979 deaths (7%) globally. This death rate is particularly alarming, as the seasonal flu generally kills far fewer than 1% of those infected[4]. The World Health Organization (WHO) has declared it the sixth public health emergency of international concern since the beginning of this century, following H1N1 (2009)[5], polio (2014), Ebola in West Africa (2014), Zika (2016), and Ebola in the Democratic Republic of Congo (2019)[6]. According to the World Health Organization (WHO) report , nearly forty million cases have been reported in more than two thousand two hundred fifteen countries worldwide, and the number of infected cases has increased gradually[7, 8].

Given the severity of COVID-19 and most countries facing with it , the WHO has called for collaborative efforts within and between countries to prevent the rapid spread of COVID-19 [9]. The WHO set the following strategic objectives for battling this disease: Limited human-to-human transmission prevents disease transmission from high-burden countries by limiting travel, identifying, isolating, and caring for patients, reducing the transmission from animal sources, developing diagnostic tools, therapeutics, and vaccines, communicating risks and events to all communities, and minimizing the social and economic impact of COVID-19 through multi-sector partnerships [2, 10]. With respect to individuals, standard recommendations to stop the spread of COVID-19 include regular hand washing, covering the mouth and nose when coughing and sneezing, and physical distancing[11].

A study conducted internationally revealed that knowledge regarding COVID-19 was adequate among most students, and the vast majority of the participants also held an optimistic attitude towards the COVID-19 [12-15]. However, findings from Saudi Arabia 31.9%[16], Middle

Eastern Arabic countries 17.0%[17], India 19.36% [18], Tanzania [19] and Uganda [20] showed that most of the community had inadequate knowledge and unfavorable attitudes towards COVId-19. Different findings also revealed that to achieve the desired control of COVID-19, adherence to control measures was essential, which is largely affected by people's knowledge, attitudes, and practices (KAP) towards the disease [13, 15, 21].

Ethiopia has applied preventive measures of COVID-19 using physical distancing, closing schools, home stay encouraging had washing practice, masks and launch of state emergency at the national level earlier to prevent and control the diseases. Despite this effort, its occurrence is increasing. Notably, in Ethiopia, as of July 17, 2020, there were a total of 8,475 cases and 148 deaths reported by the Ministry of Health and the Ethiopian Public Health Institute. Moreover, as of July 17, 2020, there are a total of 52 cases and 1 death reported in Debre Berhan, which is the study area for this article. The pandemic also puts the whole educational system in to difficult situations; predominantly, undergraduate students represented a special group that was at the ages to get independence and freedom of life but with inadequate experiences. Consequently, their KAP were suggested to be significantly affected by the pandemic, which needed to be explored. With this gap, little is known about undergraduate students' KAP. Given this fact, we aimed to assess the KAP of Debre Berhan University (DBU) students towards COVID-19.

Methods

Study design, setting, and population

A cross-sectional study was conducted from March 18–24, 2020, with undergraduate DBU students. The university is located in Debre Berhan, which are 130 km from Addis Ababa and 682 km from Bahir Dar of the Amhara regional state. According to the 2019 Registrar Office report, DBU has approximately 29,304 students pursuing regular and extension undergraduate and graduate studies. Of these, more than 11,000 are regular undergraduate students. The university has two institutes, ten colleges, and 53 departments. The source population of this study was all undergraduate students at Debre Berhan University, and the study population was all students in randomly selected collages (College of engendering, computing science, Faculty of business and economics and collage of agriculture). A table of relevant demographic details is attached as a supplementary file (**supplementary file 1).**

Sample size determination and sampling procedure (procurement procedures)

The sample size was calculated by using a single population proportion formula assuming a 95% confidence level, a 3% margin of error, and a 50% proportion for the KAP level. Since there is no study in our countries, we have used a 50% proportion[22]. After a 10% non-response rate and 1.5 design effects, the final sample size was 634. Our study sample was obtained by a two-stage cluster sampling technique. In the first stage, out of ten collages, four of them (collage of engendering, computing science, business and economics and collage of agriculture) were selected by simple random sampling, and the sample size was allocated proportionally to obtain the required sample size from each selected college. The sampling fraction is approximately equal to seven for all colleges. In the second stage, the study participants were selected from each year and sections of the selected collage using a systematic random sampling technique every 7th student from each college registrar office log-book. . After that, a dorm-to-dorm visit was used to obtain the sampled students.. Students in each selected collage that were present during the data collection period and were able to give responses were included in the study. Those who had mental or physical disabilities and could not fill out the questionnaires (unwilling to participate in to the study) were excluded from the study.

Study variables

In this study, knowledge (good/poor), attitude (positive/negative), and practice (good/poor) towards COVID-19 were the outcome variables. Socio-demographic factors, such as age, residence, sex, marital status, educational level, field of study, income, family size, and religion, and the source of information regarding COVID-19 were considered independent covariates. Knowledge of attitudes, knowledge and attitudes towards the preventive measures of the COVID-19 was also assessed.

Data collection tools

A data collection tool was developed from previous studies [23, 24] and WHO course material on emerging respiratory viruses, including COVID-19 [25]. The proposed tool was developed and validated by a multidisciplinary working group of infectious disease physicians, lecturers (Public health, nurse & environmental health professionals, and infectious diseases public health professionals. The working group regularly reviewed literature to select important characteristics and outcomes for inclusion. The development phase was conducted at Debre birhan University health science collage and the pilot validation phase was conducted at the nursing department students. We have also done a pretest from 5 % of the student. The team members formed a working group that met via telephone or video conferences at least biweekly .The questionnaire consisted of four parts: 1) socio-demographics; 2) knowledge; 3) attitudes; and 4) practices of COVID-19. Socio-demographic variables included age, sex, and marital status, and residence, year of study, average monthly income, and source of information. The second part of the survey assessed student knowledge about COVID-19, which included the symptoms of COVID-19-affected patients, transmission routes, precautions, and risk prevention. Participants were given three options per question: “Yes”, “No”, and “I don’t know”. Correct responses were given one point, while incorrect responses or “I do not know” were given zero points [15]. According to this study, “adequate knowledge” regarding COVID-19 means that the participant’s score was above the mean score on knowledge questions. Conversely, “inadequate knowledge” was assigned to students who scored below the mean score on knowledge questions. In this study, Cronbach’s alpha coefficient for the knowledge questionnaire was 0.83, indicating acceptable internal consistency [26].

Attitudes towards COVID-19 were measured with four questions. These questions used a five-point Likert-type response scale: strongly agree (5 points), agree (4 points), neutral (3 points), disagree (2 points), and strongly disagree (1 point). Using previous studies, as a baseline, we categorized agree (strongly agree” and “agree together) neutral and disagree (both disagree and strongly disagree)[20]. Subscale scores were calculated for each participant. Higher scores indicated a “favorable” attitude about COVID-19. Participant practices were assessed by yes or no questions on five specific behaviors[27] (table 4). Data were also collected through self-

administration guided by six trained data collectors. Additionally, there were no confirmed or suspected cases in the study area at the time of the data collection period.

Data quality assurance

A draft of the questionnaire was distributed to seven randomly selected faculty members to assess its readability and validity before pretesting for clarity, relevance, and acceptability. The questionnaire was initially developed in English and then translated to the local language (Amharic) by an expert and then back to English to ensure consistency. All data collectors were trained on how to properly collect data, how to maintain confidentiality, and how to ensure genuine replies to the questions. Furthermore, the principal investigator followed the data collection process on a daily basis to ensure the completeness of the questionnaires and to give further clarification to the questions when needed.

Data processing and analysis

The collected data were checked for completeness, coded, entered into Epi- Data Version 3.1, and then exported into STATA™ Version 14 software for analysis [28]. Descriptive statistics were used to describe variables (socio-demographic characteristics, participants' knowledge, attitude and practice regarding COVID-19). Binary logistic regression analyses were used to identify factors associated with KAP. The coding of outcome variables in the regression models was stated as follows. One point was given to adequate, and 0 was inadequate in the knowledge; 1 for positive, and 0 for negative in the attitude; 1 for good, and 0 for poor in the practice part. Correlations between independent variables (including the correlation between knowledge, attitude and practice) were assessed, but we did not find any correlations. The Hosmer-Lemeshow model fit-ness test was also fitted. Confounding and effect modification was evaluated by observing the regression coefficient variation greater than or equal to 15%, and multi-collinearity was checked using the variance inflation factor using a value of < 10 as a cut point. Variables with a p-value < 0.2 during bivariable analysis were used in our multivariable analyses to control for confounding effects. Factors were selected in the model using entry / standard regression**.** Adjusted odds ratios (AORs) and their 95% confidence intervals (CIs) were used to quantify the associations between variables and KAP. Adjusted odds ratios (AORs) with 95% confidence intervals (CIs) were also used to determine the strength of the association between dependent and independent variables.

Ethics consideration

Ethical clearance for this study was obtained from the Institute of Medicine and College of Health Sciences, DBU. It was approved by the University ethical approval committee with the approval number of Ref. No. DBU.R.D136/02/12. Participants also gave written informed consent prior to data collection. After that the participants were informed about their participation was completely voluntary. If they decided to take part and were still free to withdraw at any time. All information that was collected was kept strictly confidential, and personal details such as phone number and address were not revealed to people outside the research teams.

Results

Socio-demographic characteristics

A total of 546 participants completed the questionnaire with a response rate of 86.1%. The mean age of the participants was 21.7 ± 2.5 (standard deviation (SD)) years old with a range of 18–27 years old. More than half (307 (57%)) of the students were males, and nearly half (230 (42%)) of the students were in their first year. The majority of study participants (512 (93.7%)) were single, and 330 (60.4%) of the participants lived in rural areas. Additionally, most of the study participants (402 (73.6%)) heard about COVID-19 from social media (Table 1).

Table1. Socio-demographic characteristics of the study participants.

**Variables**	**Frequency**	**Percent**
**Sex**		
Female	239	43
Male	307	57
**Age group** (years)		
<20	115	21
20–24	394	72.1
≥25	38	6.9

**Marital status**<br>Single<br>Married<br>Divorced	512<br>25<br>10	93.7<br>4.5<br>1.8
**Religion**<br>Orthodox Christian<br>Muslim<br>Protestant	477<br>42<br>27	87.3<br>7.6<br>5.1
**Residence**<br>Rural<br>Urban	330<br>216	60.4<br>39.6
**Year of study**<br>First year<br>Second year<br>Third year<br>Fourth and fifth years	230<br>129<br>136<br>51	42<br>23.6<br>25<br>9.4
**Monthly pocket money/ Ethiopian Birr**<br><200<br>200–400<br>≥401	167<br>226<br>153	30.5<br>41.5<br>28
**Source of information**<br>News media<br>Social media<br>Official government websites<br>Friends and family	115<br>402<br>11<br>18	21<br>73.6<br>2<br>3.4

Note: Social media was Facebook, Twitter, WhatsApp, YouTube, Instagram, or a telegram. News media includes TV, magazines, newspapers, and radio.

Participants' knowledge regarding COVID-19

The mean COVID-19 knowledge score was 9.6 ± 1.8 with a range of 0–13. The correct answer rates for the 13 questions on the COVID-19 knowledge questionnaire were 54.4–95%. Most of the participants (403 (73.8%)) scored above the mean and were considered to have good knowledge about COVID-19. Among the 546 participants, 71.4% correctly responded that the main clinical symptoms of COVID-19 are fever, fatigue, dry cough, and shortness of breath, and the majority (95%) said currently there is no cure for COVID-19 (Table 2).

Table 2. Knowledge of the study participant towards COVID-19.

Variable	**Yes**		**No**	
	Frequency	%	Frequency	%
The main clinical symptoms of COVID-19 are fever, fatigue, dry cough, and shortness of breath	390	71.4	156	28.6
Unlike the common cold, stuffy nose, runny nose, and sneezing are less common in people infected with the COVID-19.	398	72.8	148	27.2
Currently, there is no cure for COVID-19, but early symptomatic and supportive treatment can help most patients recover from the infection.	519	95	27	5
Not all people with COVID-19 will develop a severe form of the disease. Those who are elderly, have chronic illnesses, and are obese are more likely to be severe cases.	324	59.3	222	40.7
COVID-19 is transmitted through air, contact, and feco-oral routes.	418	76.5	128	23.5
Eating or coming into contact with wild animals will lead to COVID-19 infection.	395	72.4	151	27.6
People with COVID-19 cannot infect others when a fever is not present.	249	45.6	297	54.4
The COVID-19 virus spreads via respiratory droplets from infected individuals.	350	64.1	196	35.9
Ordinary residents can wear general medical masks to prevent getting infected by the COVID-19 virus.	332	60.8	214	39.2

It is not necessary for children and young adults to take measures to prevent being infected by the COVID-19 virus.	227	41.5	319	58.5
To prevent being infected by COVID-19, individuals should avoid going to crowded places and taking public transportation.	430	78.7	116	21.3
Isolation and treatment of people who are infected with the COVID-19 virus are effective ways to reduce the spread of the virus.	445	81.5	101	18.5
People who have been in contact with someone infected with COVID-19 should self-isolate. In general, the observation period is 14 days.	473	86.7	73	13.3

Participant's attitude towards COVID-19

Nearly half (229 (42%)) of the students indicated that they have no concern of being infected with COVID-19. Most of the respondents (447 (81.8%)) agreed that COVID-19 will be successfully controlled, and the majority (458 (83.8%)) believed that Ethiopia could win the battle against the COVID-19 virus. The attitudes of students towards COVID-19 are summarized in Table 3.

Table 3. Attitudes of the DBU students towards COVID-19.

**Attitude questions**	**Agree (%)**	**Disagree (%)**	**Not sure (%)**
Do you agree that COVID-19 will be successfully controlled?	447 (81.8)	66 (12.2)	33 (6)
I have no concern of being infected with COVID-19.	229 (42)	303 (55.6)	14 (2.4)
Do you agree that washing hands with soap and water could help to prevent COVID-19 virus transmission?	415 (76)	87 (16)	44 (8)
Do you have confidence that Ethiopia can win the battle against the COVID-19 virus?	458 (83.8)	61 (11.2)	27 (5)

Participants’ practices in relation to COVID-19

The majority of the participants (91.4%) had not visited a crowded place in recent days, and approximately 74% were washing their hands after sneezing and coughing. Nearly half (284 (52%)) of the participants reported covering their mouth and nose with an elbow or tissue while coughing or sneezing. However, approximately 56% of people surveyed did not maintain a social distance of at least one meter between themselves and anyone who was coughing or sneezing (Table 4).

Table 4. Practices of DBU students with respect to COVID-19

Questions	**Yes**		**No**	
	Frequency	%	Frequency	%
In recent days, have you gone to any crowded place?	47	8.6	499	91.4
Do you wash your hands after sneezing or coughing?	404	74	142	26
Do you touch your face, nose, or mouth with your unclean hands?	377	69	169	31
Do you cover your mouth and nose with an elbow or tissue while coughing or sneezing?	284	52	262	48
In recent days, have you maintained a social distance at least one meter (three feet) between yourself and anyone who is coughing or sneezing?	240	44	306	56

.

Multivariable regression results of the binary logistic regression analyses

In our multivariable analyses, students who were 25 years old or older were 1.6 times more likely to be knowledgeable about COVID-19 than those <20 years old (AOR = 1.6, 95% CI: 1.2, 4.6). Similarly, participants who lived in urban areas were 4.3 times more knowledgeable about COVID-19 than those who resided in rural areas (AOR = 4.3, 95% CI: 2.6, 15.8). Lastly, participants who received their information on COVID-19 from the news media were 2.3 times

more likely to have a favorable attitude towards COVID-19 compared to those who received their information from friends and family (AOR = 2.3, 95% CI: 1.6, 8.7) (Table 5).

Table 5. Multivariate analyses of factors associated with knowledge and attitudes towards COVID-19 among students at DBU.

**Variables**	**Knowledge**		**AOR**	**p-value**	**Attitude**		**AOR**	**p-value**
	Good	Poor			Favorable	Not		
Sex								
Male	171 (71.5)	68 (28.5)	1.3(0.5–3.8)	0.56	157 (65.7)	82 (34.3)	1.5 (0.6–2.7)	0.47
Female	236 (76.8)	71 (23.2)	1		207 (68.5)	100 (31.5)	1	
Age (years)								
<20	71 (61.8)	44 (38.2)	1		79 (68.6)	36 (31.4)	1	
20–24	264 (67)	130 (33)	3.8 (0.6–23.3)	0.26	248 (63)	146 (37)	2.7 (0.6–13.4)	0.58
≥25	28 (73.7)	10 (26.3)	1.6 (1.2–4.6)	0.04	27 (70.3)	11 (29.7)	1.4 (0.7–6.8)	0.39
**Marital status**								
Single	336 (71.6)	176(34.4)	2.3 (0.8–9.6)	0.54	297 (58)	215 (42)	1.2 (0.6–8.8)	0.27
Married	16 (64)	9 (36)	1.2 (0.6–7.5)	0.38	14 (56)	11 (44)	1.7 (0.7–9.8)	0.49
Divorced	7 (70)	3 (30)	1		6 (60)	4 (40)	1	
**Residence**								
Rural	185 (56)	145 (44)	1		204 (62)	126 (38)	1	
Urban	188 (87)	28 (13)	4.3 (2.6–15.8)	0.01	164 (76)	52 (24)	1.7(1.1–12.4)	0.04
**Year of**								

**study**								
First	129 (56)	101 (44)	1	0.71	145 (63)	85 (34)	1	0.630.
Second	79 (61)	50 (39)	1.9 (0.7–10.3)	0.54	74 (57)	55 (43)	2.6 (0.9–13.6)	52
Third	80 (59)	56941)	1.5 (0.8–8.2)	0.03	90 (66)	46 (34)	1.7 (0.6–8.5)	0.31
Fourth and fifth years	35 (68.6)	16 (31.4)	1.4 (1.1–12.5)		32 (62.7)	19 (37.3)	1.3 (0.5–8.5)	
**Monthly pocket money**								
<200	105 (63)	62 (37)	1		90(54)	77(46)	1	
200–400	172 (76)	54 (24)	0.5 (0.2–5.6)	0.21	138(61)	88(39)	2.3(0.8-11.2)	0.46
≥401	104 (68)	49 (32)	1.6 (0.7–10.3)	0.48	96(62.8)	57(37.2)	3.2(0.7-14.6)	0.35
**Source of information**								
News media	82 (71.3)	33 (28.7)	1.8 (1.2–9.2)	0.02	87 (75.6)	28 (24.4)	2.3 (1.6–8.7)	0.01
Social media	333 (83)	69 (17)	3.4 (1.9–13.7)	0.01	314 (78)	88 (22)	2.6 (2.1–11.8)	0.01
Government websites	7 (63.6)	4 (36.4)	0.6 (0.4–4.3)	0.26	8 (72.7)	3 (27.3)	1.3 (1.1–6.2)	0.03
Friends & family	12 (66.7)	6 (33.3)	1		11 (61)	7 (39)	1	

Discussion

The KAP of a population can often determine the severity of an infectious disease outbreak. Indeed, KAP surveys have been used as important sources of data to design health interventions and public health policies. To the best of our knowledge, this is the first study in Ethiopia examining the KAP towards COVID-19 among university students. Therefore, the current study aimed to assess undergraduate student knowledge, attitudes and practices towards COVID-19 in Debre Berhan University, Ethiopia. This epidemiological survey was conducted during the very early stages of the epidemic in Ethiopia and showed that the majority of the study participants were knowledgeable about COVID-19. Supportive results were reported in China [13], Jordan [15] and India [12]. However, findings from Saudi Arabia (31.9%) [16], Middle Eastern Arabic countries (17.0%) [17], India (19.36%) [18], Tanzania [19] and Uganda [20] showed that most of the community had inadequate knowledge. This might be due to the late confirmed case report of COVID-19 in Ethiopia, which might provide time to know about the disease. Additionally, the devastating news reported about the disease and the WHO declaration of the disease as a pandemic due to its severe pathogenicity and communicability [7, 8] might also have increased the students' knowledge. **This might also indicates that regular update of students through Medias.**

Additionally, as in a study in Tanzania [19] and Jordan [15], approximately 71.4% responded correctly, indicating that the main clinical symptoms of COVID-19 are fever, fatigue, dry cough, and shortness of breath. **The high knowledge of recruited samples on the main clinical symptoms could be explained by various factors such as the seriousness of the disease and the effectiveness of different education programs in the region**.

The vast majority (95%) of participants also said that there is currently no cure for COVID-19. We also found that more than 73.6% of the participants used social media as their main source of information about COVID-19. A similar result was also reported in Jordan [15].This can help quickly disseminate important new information, relevant new scientific findings, share diagnostic, treatment, and follow-up protocols, as well as compare different approaches globally by removing geographic boundaries for the event.

In this study, the overall attitude of DBU students towards COVID-19 prevention was favorable in that they believed it would be controlled and could be prevented with good hygiene, which is in line with as tudy done at medical and non-medical university students[15]. Specifically, most of the participants (81.8%) believed that COVID-19 will eventually be controlled, and 83.8% had confidence that Ethiopia could win the battle against the virus. Similar to a finding in Syria [29], Pakistan [30] and Sudan[31], we also found that most of the study participants had poor practices regarding COVID-19 prevention. We found that approximately 48% did not cover their mouth and nose with an elbow or tissue while coughing or sneezing, and 56% did not maintain a social distance of at least one meter between themselves and anyone who was coughing or sneezing[27]. It might be due to the absence and /or poor quality of protective equipment and reservation from using protective equipment due to some discomforts. Thus, priority needs to be given to improve prevention practices parallel to awareness creation and making protective equipment available.

According to the present study, students who were 25 years old or older were 1.6 times more knowledgeable about COVID-19 than those less than 20 years old. Additionally, we found that those living in urban areas were almost four times more knowledgeable about the virus than those living in rural areas. This finding is in line with a study performed in Indonesia[32], the Nepalese[33], Sudan[31] and the South Wollo Zone[34]. This might be because those who came from urban areas have a high probability of access to different means of communication, including television and radio, which are the main channels used to teach the community about diseases.

Moreover, DBU students who heard most of their information from news media were 1.8 times more knowledgeable regarding COVID-19 compared to those who heard information from friends and family. There was a significant positive correlation between year of study and COVID-19 knowledge scores. A congruent result was reported in a study in Indonesia[32] and the South Wollo Zone[34].This might be because of easily accessible to most students at home and everywhere through the mobile internet.

With respect to the attitude of DBU students regarding COVID-19, those who lived in an urban residence had a 1.7 times more favorable attitude towards COVID-19 prevention and control compared with those living in rural areas. Moreover, participants who heard information about

the disease from government websites had a 1.3 times more favorable attitude towards COVID-19 preventive measures compared to those who heard information from friends and family. This might be because government media might provide reliable information to participants, and those who heard information from friends might hear rumors that may confuse them.

Despite our extensive efforts in reducing the possible shortcomings of this survey, this study does have certain limitations. First, the study was limited to the students of a government university only, and therefore, there is the question of the representativeness of the finding to all students. In addition, the data presented in this study are self-reported and thus may be subject to recall bias. Furthermore, the study also shares the limitations of establishing cause-effect relationships because of a cross-sectional study design.

Conclusion and recommendation

This study summarized that DBU students had moderate knowledge about COVID-19 and positive attitudes toward tackling the disease. However, most participants had poor practices with respect to containing the spread of the disease. Our study also found that urban residence, year of study, age greater than 25, and source of information about COVID-19 were significantly associated with the mean knowledge and attitude of the study participants. Importantly, nearly half of the study participants did not cover their mouth and nose with an elbow or tissue while coughing and sneezing. Moreover, more than half of the study subjects did not maintain a physical distance of at least one meter between themselves and anyone who was coughing or sneezing. Therefore, an increased effort should be made to improve the knowledge and practices of DBU students regarding COVID-19 prevention. We also recommended that the Ministry of Sciences and Higher Education [MOSHE], a zonal health bureau in collaboration with universities, implement health education programs about corona virus to university students and the wider community to address a gap in KAP towards the preventive measures of COVID-19. Social media should also work by providing timely and reliable data to students about COVID-19 KAP. A large population-based study also needs to be conducted to assess the community KAP level at large. The university in collaboration with the zonal health bureau also needs to prepare a rule a regulation on covering mouth and nose with an elbow or tissue while coughing and sneezing and to maintain a physical distancing .

Abbreviations: AORs: Adjusted odds ratios, CIs: confidence intervals, DBU: Debre Berhan University, KAP: Knowledge, Practice and Attitude, WHO: World Health Organization

Acknowledgements

The authors thank all participants involved in this study for their cooperation and support.

Supporting information

S1: A table of relevant demographic details

S2: questioner used to assess the KAP of students

References

1. **Organization WH: Operational considerations for COVID-19 management in the accommodation sector: interim guidance, 31 March 2020. In.: World Health Organization; 2020.**.
2. **The Novel Coronavirus Pneumonia Emergency Response Epidemiology Team. The epidemiological characteristics of an outbreak of 2019 novel coronavirus diseases (COVID-19) in China. Chin J Epidemiol. 2020; 41: 145-51.**
3. **Chen N, Zhou M, Dong X, Qu J, Gong F, Han Y, et al. Epidemiological and clinical characteristics of 99 cases of 2019 novel coronavirus pneumonia in Wuhan, China: a descriptive study. Lancet. 2020; 395: 507-13.**
4. **Organization WH: Novel Coronavirus (2019-nCoV): situation report, 3. 2020.**
5. **M.P. Girard, J.S. Tam, O.M. Assossou, M.P. Kieny. The 2009 A (H1N1) influenza virus pandemic: A review. Vaccine 2010, 28 (31), 4895–4902. .**
6. **J. Kaner, S. Schaack. Understanding Ebola: the 2014 epidemic. Global. Health 2016, 12 (1), 53. .**
7. **Wu P, Hao X, Lau EH, Wong JY, Leung KS, Wu JT, Cowling BJ, Leung GM: Real-time tentative assessment of the epidemiological characteristics of novel coronavirus infections in Wuhan, China, as at 22 January 2020. *Eurosurveillance* 2020, 25(3):2000044**
8. **Thompson R: Pandemic potential of 2019-nCoV. The Lancet Infectious Diseases 2020, 20(3):280**.
9. Team EE: **Note from the editors: World Health Organization declares novel coronavirus (2019-nCoV) sixth public health emergency of international concern**. *Eurosurveillance* 2020, **25**(5).
10. Uddin MN, Alam B, Islam SS, Arif M, Alam MM, Kabir SL: **Impact of COVID-19 on food safety and security in low and middle income countries**. *Asian Journal of Medical and Biological Research* 2020, **6**(2):130-137.
11. **Organization WH: Considerations in adjusting public health and social measures in the context ofCOVID-19: interim guidance, 16 April 2020. In.: World Health Organization; 2020.**
12. Gupta L, Agarwal V, Davalbhakta S, Agarwal V, Misra D: **A survey-based study on the knowledge, attitude, and the practices pertaining to the 2019 novel Corona Virus infection amongst undergraduate medical students in India**. *medRxiv* 2020.
13. Peng Y, Pei C, Zheng Y, Wang J, Zhang K, Zheng Z, Zhu P: **Knowledge, attitude and practice associated with COVID-19 among university students: a cross-sectional survey in China**. 2020.
14. Gallè F, Sabella EA, Da Molin G, De Giglio O, Caggiano G, Di Onofrio V, Ferracuti S, Montagna MT, Liguori G, Orsi GB: **Understanding Knowledge and Behaviors Related to CoViD–19 Epidemic in Italian Undergraduate Students: The EPICO Study**. *International Journal of Environmental Research and Public Health* 2020, **17**(10):3481.
15. Olaimat AN, Aolymat I, Shahbaz HM, Holley RA: **Knowledge and Information Sources About COVID-19 Among University Students in Jordan: A Cross-Sectional Study**. *Frontiers in Public Health* 2020, **8**:254.
16. Baig M, Jameel T, Alzahrani SH, Mirza AA, Gazzaz ZJ, Ahmad T, Baig F, Almurashi SH: **Predictors of misconceptions, knowledge, attitudes, and practices of COVID-19 pandemic among a sample of Saudi population and its impact: a cross-sectional study**. *medRxiv* 2020.
17. Naser AY, Dahmash EZ, Alwafi H, Alsairafi ZK, Al Rajeh AM, Alhartani YJ, Turkistani FM, Alyami HS: **Knowledge and practices towards COVID-19 during its outbreak: a multinational cross-sectional study**. *medRxiv* 2020.
18. Tomar BS, Singh P, Nathiya D, Suman S, Raj P, Tripathi S, Chauhan DS: **Indian communitys Knowledge, Attitude & Practice towards COVID-19**. *medRxiv* 2020.

19. Rugarabamu S, Ibrahim M, Byanaku A: **Knowledge, attitudes, and practices (KAP) towards COVID-19: A quick online cross-sectional survey among Tanzanian residents**.
20. Ssebuufu R, Sikakulya F, Binezero SM, Wasingya L, Nganza SK, Ibrahim B, Kyamanywa P: **Awareness, knowledge, attitude and practice towards measures for prevention of the spread of COVID-19 in the Ugandans: A nationwide online cross-sectional Survey**. *medRxiv* 2020.
21. Al-Hazmi A, Gosadi I, Somily A, Alsubaie S, Saeed AB: **Knowledge, attitude and practice of secondary schools and university students toward Middle East Respiratory Syndrome epidemic in Saudi Arabia: A cross-sectional study**. *Saudi journal of biological sciences* 2018, **25**(3):572-577.
22. Suresh K, Chandrashekara S: **Sample size estimation and power analysis for clinical research studies**. *Journal of human reproductive sciences* 2012, **5**(1):7.
23. Bhagavathula AS, Aldhaleei WA, Rahmani J, Mahabadi MA, Bandari DK: **Novel Coronavirus (COVID-19) Knowledge and Perceptions: A Survey on Healthcare workers**. *medRxiv* 2020.
24. Zhong B-L, Luo W, Li H-M, Zhang Q-Q, Liu X-G, Li W-T, Li Y: **Knowledge, attitudes, and practices towards COVID-19 among Chinese residents during the rapid rise period of the COVID-19 outbreak: a quick online cross-sectional survey**. *Int J Biol Sci* 2020, **16**(10):1745-1752.
25. orld health organization. Emerging respiratory viruses iC-m.
26. Taber KS: **The use of Cronbach's alpha when developing and reporting research instruments in science education**. *Research in Science Education* 2018, **48**(6):1273-1296.
27. Maheshwari S, Gupta PK, Sinha R, Rawat P: **Knowledge, attitude, and practice towards coronavirus disease 2019 (COVID-19) among medical students: A cross-sectional study**. *Journal of Acute Disease* 2020, **9**(3):100.
28. StataCorp L: **Stata statistical software (version release 14)**. *College Station, TX: Author* 2015.
29. **Al Ahdab S: Knowledge, Attitudes and Practices (KAP) towards pandemic COVID-19 among Syrians. 2020.**
30. **Saqlain M, Ahmed A, Gulzar A, Naz S, Munir MM, Ahmed Z, Kamran S: Public Knowledge and Practices regarding COVID-19: A cross-sectional survey from Pakistan. medRxiv 2020.**
31. **Mohamed A, Elhassan E, Mohamed AO, Mohammed AA, Mahgoop MA, Sharif ME, Bashir MI, Abderlahim RB, Idriss WI, Malik EM: Knowledge, attitude and practice of the Sudanese people towards COVID-19: An online survey. 2020.**
32. **Saefi M, Fauzi A, Kristiana E, Adi WC, Muchson M, et al. Survey data of COVID-19-related Knowledge, Attitude, and Practices among Indonesian Undergraduate Students. Data in Brief 2020:105855.**
33. **Paudel S, Shrestha P, Karmacharya I, Pathak OK. Knowledge, attitude, and practices (KAP) towards COVID-19 among Nepalese residents during the COVID-19 outbreak: An online crosssectional study. 2020.**
34. Tadesse AW, Melese N, Eshetie S, Chane M, Ali A: **Knowledge, Attitude, and Practice and Associated Factors towards COVID-19 among College Students in Amhara Region, Ethiopia; A Cross-Sectional Study**. 2020.

Click here to access/download
Supporting Information
supplemetary 2.docx

Click here to access/download
Supporting Information
supplemetary file 1.docx

Revised Manuscript with Track Changes

Assessment of undergraduate student knowledge, attitude, and practices, towards COVID-19 in Debre Berhan University, Ethiopia

REMOVE comma

Yared Asmare Aynalem[1*] Tadess Yirga Akalu[2], Birhan Gebresellassie[1], Nigussie Tadesse Sharew[1,] Hilina Ketema Assefa[1],Wondimeneh Shibabaw Shiferaw[1]

[1]Department of Nursing, College of Health Science, Debre Berhan University, Debre Berhan, Ethiopia

[2]Department of Nursing, College of Health Science, Debre Markos University, Debre Markos, Ethiopia

[*]Corresponding author: yaredasmare123@gmail.com

Abstract

Background: Novel coronavirus-2019 (COVID-19) is a highly infectious disease that has caused a global pandemic. As of July 2020, there were 8,475 confirmed cases of COVID-19 in Ethiopia, and a total of 52 cases and 1 death were reported in Debre Berhan where this study was conducted. Under these conditions, we sought to assess what undergraduate students at Debre Berhan University knew about COVID-19 and how it shaped their attitudes and practices regarding this disease.

Objective: The aim of the current study was to assess undergraduate student knowledge, attitudes and practices towards COVID-19 in Debre Berhan University, Ethiopia.

Methods: A cross-sectional survey was conducted from March 18–24, 2020 among undergraduate students at Debre Berhan University. A two-stage cluster sampling technique was employed with a total sample size of 634. Proportional allocation of samples was used to the randomly selected colleges, and a systematic random sampling technique was employed to recruit the students. The data were checked for completeness, coded, entered into Epi-Data VS 3.1, and then exported into STATA™ Version 14 software for analysis. Descriptive statistics were conducted. Binary logistic regression analyses were used to identify factors. Factors were selected with the entry method. Adjusted odds ratios (AORs) and their 95% confidence intervals (CIs) were used to assess the associations between variables and knowledge, attitude and practices (KAP).

Results: From a total of 546 included participants, more than half of them, 307 (57%) were males. Seventy-three percent of them heard about novel coronavirus from social media. In this study, 73.8% of the participants were knowledgeable, and their overall attitude was favorable. Approximately 71.4% correctly responded that the main clinical symptoms of COVID-19 are fever, fatigue, dry cough, and shortness of breath. Nearly half, 229 (42%) of the students agreed that they had no concern of being infected with COVID-19. Moreover, most participants showed poor practices; more than half of the study subjects were not maintaining a physical distance. In multivariable analyses, people 25 and older (AOR = 1.6, 95% CI; 1.2, 4.6) and those who lived in urban areas (AOR = 4.3, 95% CI; 2.6, 15.8) were significantly associated with being knowledgeable about COVID-19. Furthermore, we found that the source of information about

COVID-19 (AOR = 2.3, 95% CI; 1.6, 8.7) was significantly associated with the attitude undergraduate students had about COVID-19. PLEASE CLARIFY

Conclusion: The undergraduate students at Debre Berhan University were moderately knowledgeable about COVID-19 and had an optimistic attitude towards its resolution. However, this optimism may lead to poor public health practices within this community. Therefore, greater efforts need to be made through more comprehensive and directed actions and awareness campaigns to increase the knowledge, attitude and practice of the students.

Background

The novel coronavirus-2019 (COVID-19) is a newly discovered infectious disease that can cause severe illness in humans[1]. It was first detected in December 2019 in Wuhan, China, and quickly spread around the world. Fever, dry cough, fatigue, myalgia, and dyspnea are considered the main clinical symptoms of this disease. In China, 18.5% of the patients with COVID-19 developed acute respiratory distress syndrome, septic shock, metabolic acidosis, and bleeding. In more severe cases, it can cause pneumonia, severe acute respiratory syndrome, kidney failure, and even death[2, 3].

As of July 17, 2020, there were 13,961,427 confirmed cases and 592,979 deaths (7%) globally. This death rate is particularly alarming, as the seasonal flu generally kills far fewer than 1% of those infected[4]. The World Health Organization (WHO) has declared it the sixth public health emergency of international concern since the beginning of this century, following H1N1 (2009)[5], polio (2014), Ebola in West Africa (2014), Zika (2016), and Ebola in the Democratic Republic of Congo (2019)[6]. According to the World Health Organization (WHO) report , nearly forty million cases have been reported in more than two thousand two hundred fifteen countries worldwide, and the number of infected cases has increased gradually[7, 8].

Given the severity of COVID-19 and most countries facing with it , the WHO has called for collaborative efforts within and between countries to prevent the rapid spread of COVID-19 [9]. The WHO set the following strategic objectives for battling this disease: Limited human-to-human transmission prevents disease transmission from high-burden countries by limiting travel, identifying, isolating, and caring for patients, reducing the transmission from animal sources, developing diagnostic tools, therapeutics, and vaccines, communicating risks and events to all communities, and minimizing the social and economic impact of COVID-19 through multi-sector partnerships [2, 10]. With respect to individuals, standard recommendations to stop the spread of COVID-19 include regular hand washing, covering the mouth and nose when coughing and sneezing, and physical distancing[11].

A study conducted internationally revealed that knowledge regarding COVID-19 was adequate among most students, and the vast majority of the participants also held an optimistic attitude towards the COVID-19 [12-15]. However, findings from Saudi Arabia 31.9%[16], Middle

Eastern Arabic countries 17.0%[17], India 19.36% [18], Tanzania [19] and Uganda [20] showed that most of the community had inadequate knowledge and unfavorable attitudes towards COVId-19. Different findings also revealed that to achieve the desired control of COVID-19, adherence to control measures was essential, which is largely affected by people's knowledge, attitudes, and practices (KAP) towards the disease [13, 15, 21].

Ethiopia has applied preventive measures of COVID-19 using physical distancing, closing schools, home stay encouraging had washing practice, masks and launch of state emergency at the national level earlier to prevent and control the diseases. Despite this effort, its occurrence is increasing. Notably, in Ethiopia, as of July 17, 2020, there were a total of 8,475 cases and 148 deaths reported by the Ministry of Health and the Ethiopian Public Health Institute. Moreover, as of July 17, 2020, there are a total of 52 cases and 1 death reported in Debre Berhan, which is the study area for this article. The pandemic also puts the whole educational system in to difficult situations; predominantly, undergraduate students represented a special group that was at the ages to get independence and freedom of life but with inadequate experiences. Consequently, their KAP were suggested to be significantly affected by the pandemic, which needed to be explored. With this gap, little is known about undergraduate students' KAP. Given this fact, we aimed to assess the KAP of Debre Berhan University (DBU) students towards COVID-19.

Methods

Study design, setting, and population

A cross-sectional study was conducted from March 18–24, 2020, with undergraduate DBU students. The university is located in Debre Berhan, which are 130 km from Addis Ababa and 682 km from Bahir Dar of the Amhara regional state. According to the 2019 Registrar Office report, DBU has approximately 29,304 students pursuing regular and extension undergraduate and graduate studies. Of these, more than 11,000 are regular undergraduate students. The university has two institutes, ten colleges, and 53 departments. The source population of this study was all undergraduate students at Debre Berhan University, and the study population was all students in randomly selected collages (College of engendering, computing science, Faculty of business and economics and collage of agriculture). A table of relevant demographic details is attached as a supplementary file (**supplementary file 1).**

Sample size determination and sampling procedure (procurement procedures)

The sample size was calculated by using a single population proportion formula assuming a 95% confidence level, a 3% margin of error, and a 50% proportion for the KAP level. Since there is no study in our countries, we have used a 50% proportion[22]. After a 10% non-response rate and 1.5 design effects, the final sample size was 634. Our study sample was obtained by a two-stage cluster sampling technique. In the first stage, out of ten collages, four of them (collage of engendering, computing science, business and economics and collage of agriculture) were selected by simple random sampling, and the sample size was allocated proportionally to obtain the required sample size from each selected college. The sampling fraction is approximately equal to seven for all colleges. In the second stage, the study participants were selected from each year and sections of the selected collage using a systematic random sampling technique every 7th student from each college registrar office log-book. . After that, a dorm-to-dorm visit was used to obtain the sampled students.. Students in each selected collage that were present during the data collection period and were able to give responses were included in the study. Those who had mental or physical disabilities and could not fill out the questionnaires (unwilling to participate in to the study) were excluded from the study.

Study variables

In this study, knowledge (good/poor), attitude (positive/negative), and practice (good/poor) towards COVID-19 were the outcome variables. Socio-demographic factors, such as age, residence, sex, marital status, educational level, field of study, income, family size, and religion, and the source of information regarding COVID-19 were considered independent covariates. Knowledge of attitudes, knowledge and attitudes towards the preventive measures of the COVID-19 was also assessed.

Data collection tools

A data collection tool was developed from previous studies [23, 24] and WHO course material on emerging respiratory viruses, including COVID-19 [25]. The proposed tool was developed and validated by a multidisciplinary working group of infectious disease physicians, lecturers (Public health, nurse & environmental health professionals, and infectious diseases public health professionals. The working group regularly reviewed literature to select important characteristics and outcomes for inclusion. The development phase was conducted at Debre birhan University health science collage and the pilot validation phase was conducted at the nursing department students. We have also done a pretest from 5 % of the student. The team members formed a working group that met via telephone or video conferences at least biweekly .The questionnaire consisted of four parts: 1) socio-demographics; 2) knowledge; 3) attitudes; and 4) practices of COVID-19. Socio-demographic variables included age, sex, and marital status, and residence, year of study, average monthly income, and source of information. The second part of the survey assessed student knowledge about COVID-19, which included the symptoms of COVID-19-affected patients, transmission routes, precautions, and risk prevention. Participants were given three options per question: "Yes", "No", and "I don't know". Correct responses were given one point, while incorrect responses or "I do not know" were given zero points [15]. According to this study, "adequate knowledge" regarding COVID-19 means that the participant's score was above the mean score on knowledge questions. Conversely, "inadequate knowledge" was assigned to students who scored below the mean score on knowledge questions. In this study, Cronbach's alpha coefficient for the knowledge questionnaire was 0.83, indicating acceptable internal consistency [26].

Attitudes towards COVID-19 were measured with four questions. These questions used a five-point Likert-type response scale: strongly agree (5 points), agree (4 points), neutral (3 points), disagree (2 points), and strongly disagree (1 point). Using previous studies, as a baseline, we categorized agree (strongly agree" and "agree together) neutral and disagree (both disagree and strongly disagree)[20]. Subscale scores were calculated for each participant. Higher scores indicated a "favorable" attitude about COVID-19. Participant practices were assessed by yes or no questions on five specific behaviors[27] (table 4). Data were also collected through self-administration guided by six trained data collectors. Additionally, there were no confirmed or suspected cases in the study area at the time of the data collection period.

Data quality assurance

A draft of the questionnaire was distributed to seven randomly selected faculty members to assess its readability and validity before pretesting for clarity, relevance, and acceptability. The questionnaire was initially developed in English and then translated to the local language (Amharic) by an expert and then back to English to ensure consistency. All data collectors were trained on how to properly collect data, how to maintain confidentiality, and how to ensure genuine replies to the questions. Furthermore, the principal investigator followed the data collection process on a daily basis to ensure the completeness of the questionnaires and to give further clarification to the questions when needed.

Data processing and analysis

The collected data were checked for completeness, coded, entered into Epi- Data Version 3.1, and then exported into STATA™ Version 14 software for analysis [28]. Descriptive statistics were used to describe variables (socio-demographic characteristics, participants' knowledge, attitude and practice regarding COVID-19). Binary logistic regression analyses were used to identify factors associated with KAP. The coding of outcome variables in the regression models was stated as follows. One point was given to adequate, and 0 was inadequate in the knowledge; 1 for positive, and 0 for negative in the attitude; 1 for good, and 0 for poor in the practice part. Correlations between independent variables (including the correlation between knowledge, attitude and practice) were assessed, but we did not find any correlations. The Hosmer-Lemeshow model fit-ness test was also fitted. Confounding and effect modification was evaluated by observing the regression coefficient variation greater than or equal to 15%, and multi-collinearity was checked using the variance inflation factor using a value of < 10 as a cut point. Variables with a p-value < 0.2 during bivariable analysis were used in our multivariable analyses to control for confounding effects. Factors were selected in the model using entry / standard regression**.** Adjusted odds ratios (AORs) and their 95% confidence intervals (CIs) were used to quantify the associations between variables and KAP. Adjusted odds ratios (AORs) with 95% confidence intervals (CIs) were also used to determine the strength of the association between dependent and independent variables.

Ethics consideration

Ethical clearance for this study was obtained from the Institute of Medicine and College of Health Sciences, DBU. It was approved by the University ethical approval committee with the approval number of Ref. No. DBU.R.D136/02/12. Participants also gave written informed consent prior to data collection. After that the participants were informed about their participation was completely voluntary. If they decided to take part and were still free to withdraw at any time. All information that was collected was kept strictly confidential, and personal details such as phone number and address were not revealed to people outside the research teams.

Results

Socio-demographic characteristics

A total of 546 participants completed the questionnaire with a response rate of 86.1%. The mean age of the participants was 21.7 ± 2.5 (standard deviation (SD)) years old with a range of 18–27 years old. More than half (307 (57%)) of the students were males, and nearly half (230 (42%)) of the students were in their first year. The majority of study participants (512 (93.7%)) were single, and 330 (60.4%) of the participants lived in rural areas. Additionally, most of the study participants (402 (73.6%)) heard about COVID-19 from social media (Table 1).

Table1. Socio-demographic characteristics of the study participants.

**Variables**	**Frequency**	**Percent**
**Sex**		
Female	239	43
Male	307	57
**Age group** (years)		
<20	115	21
20–24	394	72.1
≥25	38	6.9
**Marital status**		
	512	93.7

Single	25	4.5
Married	10	1.8
Divorced		
**Religion**		
Orthodox Christian	477	87.3
Muslim	42	7.6
Protestant	27	5.1
**Residence**		
Rural	330	60.4
Urban	216	39.6
**Year of study**		
First year	230	42
Second year	129	23.6
Third year	136	25
Fourth and fifth years	51	9.4
**Monthly pocket money/ Ethiopian Birr**		
<200	167	30.5
200–400	226	41.5
≥401	153	28
**Source of information**		
News media	115	21
Social media	402	73.6
Official government websites	11	2
Friends and family	18	3.4

Note: Social media was Facebook, Twitter, WhatsApp, YouTube, Instagram, or a telegram. News media includes TV, magazines, newspapers, and radio.

Participants' knowledge regarding COVID-19

The mean COVID-19 knowledge score was 9.6 ± 1.8 with a range of 0–13. The correct answer rates for the 13 questions on the COVID-19 knowledge questionnaire were 54.4–95%. Most of the participants (403 (73.8%)) scored above the mean and were considered to have good knowledge about COVID-19. Among the 546 participants, 71.4% correctly responded that the main clinical symptoms of COVID-19 are fever, fatigue, dry cough, and shortness of breath, and the majority (95%) said currently there is no cure for COVID-19 (Table 2).

Table 2. Knowledge of the study participant towards COVID-19.

Variable	**Yes**		**No**	
	Frequency	%	Frequency	%
The main clinical symptoms of COVID-19 are fever, fatigue, dry cough, and shortness of breath	390	71.4	156	28.6
Unlike the common cold, stuffy nose, runny nose, and sneezing are less common in people infected with the COVID-19.	398	72.8	148	27.2
Currently, there is no cure for COVID-19, but early symptomatic and supportive treatment can help most patients recover from the infection.	519	95	27	5
Not all people with COVID-19 will develop a severe form of the disease. Those who are elderly, have chronic illnesses, and are obese are more likely to be severe cases.	324	59.3	222	40.7
COVID-19 is transmitted through air, contact, and feco-oral routes.	418	76.5	128	23.5
Eating or coming into contact with wild animals will lead to COVID-19 infection.	395	72.4	151	27.6
People with COVID-19 cannot infect others when a fever is not present.	249	45.6	297	54.4
The COVID-19 virus spreads via respiratory droplets from infected individuals.	350	64.1	196	35.9
Ordinary residents can wear general medical masks to prevent getting infected by the COVID-19 virus.	332	60.8	214	39.2
It is not necessary for children and young adults to take measures to prevent being infected by the COVID-19 virus.	227	41.5	319	58.5

To prevent being infected by COVID-19, individuals should avoid going to crowded places and taking public transportation.	430	78.7	116	21.3
Isolation and treatment of people who are infected with the COVID-19 virus are effective ways to reduce the spread of the virus.	445	81.5	101	18.5
People who have been in contact with someone infected with COVID-19 should self-isolate. In general, the observation period is 14 days.	473	86.7	73	13.3

Participant's attitude towards COVID-19

Nearly half (229 (42%)) of the students indicated that they have no concern of being infected with COVID-19. Most of the respondents (447 (81.8%)) agreed that COVID-19 will be successfully controlled, and the majority (458 (83.8%)) believed that Ethiopia could win the battle against the COVID-19 virus. The attitudes of students towards COVID-19 are summarized in Table 3.

Table 3. Attitudes of the DBU students towards COVID-19.

**Attitude questions**	**Agree (%)**	**Disagree (%)**	**Not sure (%)**
Do you agree that COVID-19 will be successfully controlled?	447 (81.8)	66 (12.2)	33 (6)
I have no concern of being infected with COVID-19.	229 (42)	303 (55.6)	14 (2.4)
Do you agree that washing hands with soap and water could help to prevent COVID-19 virus transmission?	415 (76)	87 (16)	44 (8)
Do you have confidence that Ethiopia can win the battle against the COVID-19 virus?	458 (83.8)	61 (11.2)	27 (5)

Participants' practices in relation to COVID-19

The majority of the participants (91.4%) had not visited a crowded place in recent days, and approximately 74% were washing their hands after sneezing and coughing. Nearly half (284 (52%)) of the participants reported covering their mouth and nose with an elbow or tissue while coughing or sneezing. However, approximately 56% of people surveyed did not maintain a social distance of at least one meter between themselves and anyone who was coughing or sneezing (Table 4).

Table 4. Practices of DBU students with respect to COVID-19

Questions	**Yes**		**No**	
	Frequency	%	Frequency	%
In recent days, have you gone to any crowded place?	47	8.6	499	91.4
Do you wash your hands after sneezing or coughing?	404	74	142	26
Do you touch your face, nose, or mouth with your unclean hands?	377	69	169	31
Do you cover your mouth and nose with an elbow or tissue while coughing or sneezing?	284	52	262	48
In recent days, have you maintained a social distance at least one meter (three feet) between yourself and anyone who is coughing or sneezing?	240	44	306	56

.

Multivariable regression results of the binary logistic regression analyses

In our multivariable analyses, students who were 25 years old or older were 1.6 times more likely to be knowledgeable about COVID-19 than those <20 years old (AOR = 1.6, 95% CI: 1.2, 4.6). Similarly, participants who lived in urban areas were 4.3 times more knowledgeable about COVID-19 than those who resided in rural areas (AOR = 4.3, 95% CI: 2.6, 15.8). Lastly, participants who received their information on COVID-19 from the news media were 2.3 times more likely to have a favorable attitude towards COVID-19 compared to those who received their information from friends and family (AOR = 2.3, 95% CI: 1.6, 8.7) (Table 5).

Table 5. Multivariate analyses of factors associated with knowledge and attitudes towards COVID-19 among students at DBU.

Variables	Knowledge		AOR	p-value	Attitude		AOR	p-value
	Good	Poor			Favorable	Not		
Sex								
Male	171 (71.5)	68 (28.5)	1.3(0.5–3.8)	0.56	157 (65.7)	82 (34.3)	1.5 (0.6–2.7)	0.47
Female	236 (76.8)	71 (23.2)	1		207 (68.5)	100 (31.5)	1	
Age (years)								
<20	71 (61.8)	44 (38.2)	1		79 (68.6)	36 (31.4)	1	
20–24	264 (67)	130 (33)	3.8 (0.6–23.3)	0.26	248 (63)	146 (37)	2.7 (0.6–13.4)	0.58
≥25	28 (73.7)	10 (26.3)	1.6 (1.2–4.6)	0.04	27 (70.3)	11 (29.7)	1.4 (0.7–6.8)	0.39
**Marital status**								
Single	336 (71.6)	176(34.4)	2.3 (0.8–9.6)	0.54	297 (58)	215 (42)	1.2 (0.6–8.8)	0.27
Married	16 (64)	9 (36)		0.38	14 (56)	11 (44)	1.7 (0.7–9.8)	0.49
Divorced	7 (70)	3 (30)	1.2 (0.6–7.5)		6 (60)	4 (40)	1	
			1					
**Residence**								
Rural	185 (56)	145 (44)	1	0.01	204 (62)	126 (38)	1	
Urban	188 (87)	28 (13)	4.3 (2.6–15.8)		164 (76)	52 (24)	1.7(1.1–12.4)	0.04
**Year of study**								
First	129 (56)	101 (44)	1	0.71	145 (63)	85 (34)	1	0.630.
Second	79 (61)	50 (39)	1.9 (0.7–10.3)		74 (57)	55 (43)	2.6 (0.9–13.6)	

Third	80 (59)	56941)	1.5 (0.8–8.2)	0.54	90 (66)	46 (34)	1.7 (0.6–8.5)	52
Fourth and fifth years	35 (68.6)	16 (31.4)	1.4 (1.1–12.5)	0.03	32 (62.7)	19 (37.3)	1.3 (0.5–8.5)	0.31
**Monthly pocket money**								
<200	105 (63)	62 (37)	1		90(54)	77(46)	1	
200–400	172 (76)	54 (24)	0.5 (0.2–5.6)	0.21	138(61)	88(39)	2.3(0.8-11.2)	0.46
≥401	104 (68)	49 (32)	1.6 (0.7–10.3)	0.48	96(62.8)	57(37.2)	3.2(0.7-14.6)	0.35
**Source of information**								
News media	82 (71.3)	33 (28.7)	1.8 (1.2–9.2)	0.02	87 (75.6)	28 (24.4)	2.3 (1.6–8.7)	0.01
Social media	333 (83)	69 (17)	3.4 (1.9–13.7)	0.01	314 (78)	88 (22)	2.6 (2.1–11.8)	0.01
Government websites	7 (63.6)	4 (36.4)	0.6 (0.4–4.3)	0.26	8 (72.7)	3 (27.3)	1.3 (1.1–6.2)	0.03
Friends & family	12 (66.7)	6 (33.3)	1		11 (61)	7 (39)	1	

Discussion

The KAP of a population can often determine the severity of an infectious disease outbreak. Indeed, KAP surveys have been used as important sources of data to design health interventions and public health policies. To the best of our knowledge, this is the first study in Ethiopia examining the KAP towards COVID-19 among university students. ~~Therefore, the current study~~

WHAT DOES IT MEAN?

aimed to assess undergraduate student knowledge, attitudes and practices towards COVID-19 in Debre Berhan University, Ethiopia. This epidemiological survey was conducted during the very early stages of the epidemic in Ethiopia and showed that the majority of the study participants were knowledgeable about COVID-19. Supportive results were reported in China [13], Jordan [15] and India [12]. However, findings from Saudi Arabia (31.9%) [16], Middle Eastern Arabic countries (17.0%) [17], India (19.36%) [18], Tanzania [19] and Uganda [20] showed that most of the community had inadequate knowledge. This might be due to the late confirmed case report of COVID-19 in Ethiopia, which might provide time to know about the disease. Additionally, the devastating news reported about the disease and the WHO declaration of the disease as a pandemic due to its severe pathogenicity and communicability [7, 8] might also have increased the students' knowledge. **This might also indicates that regular update of students through Medias.**

THIS SENTENCE NEEDS REFERENCE + EXPANSION

Additionally, as in a study in Tanzania [19] and Jordan [15], approximately 71.4% responded correctly, indicating that the main clinical symptoms of COVID-19 are fever, fatigue, dry cough, and shortness of breath. **The high knowledge of recruited samples on the main clinical symptoms could be explained by various factors such as the seriousness of the disease and the effectiveness of different education programs in the region**.

HAVE THERE BEEN PROGRAMS FOR STUDENTS??

The vast majority (95%) of participants also said that there is currently no cure for COVID-19. We also found that more than 73.6% of the participants used social media as their main source of information about COVID-19. A similar result was also reported in Jordan [15].This can help quickly disseminate important new information, relevant new scientific findings, share diagnostic, treatment, and follow-up protocols, as well as compare different approaches globally by removing geographic boundaries for the event.

In this study, the overall attitude of DBU students towards COVID-19 prevention was favorable in that they believed it would be controlled and could be prevented with good hygiene, which is in line with as tudy done at medical and non-medical university students[15]. Specifically, most of the participants (81.8%) believed that COVID-19 will eventually be controlled, and 83.8% had confidence that Ethiopia could win the battle against the virus. Similar to a finding in Syria [29], Pakistan [30] and Sudan[31], we also found that most of the study participants had poor practices regarding COVID-19 prevention. We found that approximately 48% did not cover their

mouth and nose with an elbow or tissue while coughing or sneezing, and 56% did not maintain a social distance of at least one meter between themselves and anyone who was coughing or sneezing[27]. It might be due to the absence and /or poor quality of protective equipment and reservation from using protective equipment due to some discomforts. Thus, priority needs to be given to improve prevention practices parallel to awareness creation and making protective equipment available.

According to the present study, students who were 25 years old or older were 1.6 times more knowledgeable about COVID-19 than those less than 20 years old. Additionally, we found that those living in urban areas were almost four times more knowledgeable about the virus than those living in rural areas. This finding is in line with a study performed in Indonesia[32], the Nepalese[33], Sudan[31] and the South Wollo Zone[34]. This might be because those who came from urban areas have a high probability of access to different means of communication, including television and radio, which are the main channels used to teach the community about diseases. needs reference

Moreover, DBU students who heard most of their information from news media were 1.8 times more knowledgeable regarding COVID-19 compared to those who heard information from friends and family. There was a significant positive correlation between year of study and COVID-19 knowledge scores. A congruent result was reported in a study in Indonesia[32] and the South Wollo Zone[34].This might be because of easily accessible to most students at home and everywhere through the mobile internet. need to add reference

With respect to the attitude of DBU students regarding COVID-19 those who lived in an urban residence had a 1.7 times more favorable attitude towards COVID-19 prevention and control compared with those living in rural areas. Moreover, participants who heard information about the disease from government websites had a 1.3 times more favorable attitude towards COVID-19 preventive measures compared to those who heard information from friends and family. This might be because government media might provide reliable information to participants, and those who heard information from friends might hear rumors that may confuse them.

Despite our extensive efforts in reducing the possible shortcomings of this survey, this study does have certain limitations. First, the study was limited to the students of a government university only, and therefore, there is the question of the representativeness of the finding to all

students. In addition, the data presented in this study are self-reported and thus may be subject to recall bias. Furthermore, the study also shares the limitations of establishing cause-effect relationships because of a cross-sectional study design.

Conclusion and recommendation

This study summarized that DBU students had moderate knowledge about COVID-19 and positive attitudes toward tackling the disease. However, most participants had poor practices with respect to containing the spread of the disease. Our study also found that urban residence, year of study, age greater than 25, and source of information about COVID-19 were significantly associated with the mean knowledge and attitude of the study participants. Importantly, nearly half of the study participants did not cover their mouth and nose with an elbow or tissue while coughing and sneezing. Moreover, more than half of the study subjects did not maintain a physical distance of at least one meter between themselves and anyone who was coughing or sneezing. Therefore, an increased effort should be made to improve the knowledge and practices of DBU students regarding COVID-19 prevention. We also recommended that the Ministry of Sciences and Higher Education [MOSHE], a zonal health bureau in collaboration with universities, implement health education programs about corona virus to university students and the wider community to address a gap in KAP towards the preventive measures of COVID-19. Social media should also work by providing timely and reliable data to students about COVID-19 KAP. A large population-based study also needs to be conducted to assess the community KAP level at large. The university in collaboration with the zonal health bureau also needs to prepare a rule a regulation on covering mouth and nose with an elbow or tissue while coughing and sneezing and to maintain a physical distancing .

Abbreviations: AORs: Adjusted odds ratios, CIs: confidence intervals, DBU: Debre Berhan University, KAP: Knowledge, Practice and Attitude, WHO: World Health Organization

Acknowledgements

The authors thank all participants involved in this study for their cooperation and support.

Supporting information

S1: A table of relevant demographic details

S2: questioner used to assess the KAP of students

References

1. **Organization WH: Operational considerations for COVID-19 management in the accommodation sector: interim guidance, 31 March 2020. In.: World Health Organization; 2020.**.
2. **The Novel Coronavirus Pneumonia Emergency Response Epidemiology Team. The epidemiological characteristics of an outbreak of 2019 novel coronavirus diseases (COVID-19) in China. Chin J Epidemiol. 2020; 41: 145-51.**
3. **Chen N, Zhou M, Dong X, Qu J, Gong F, Han Y, et al. Epidemiological and clinical characteristics of 99 cases of 2019 novel coronavirus pneumonia in Wuhan, China: a descriptive study. Lancet. 2020; 395: 507-13.**
4. **Organization WH: Novel Coronavirus (2019-nCoV): situation report, 3. 2020.**
5. **M.P. Girard, J.S. Tam, O.M. Assossou, M.P. Kieny. The 2009 A (H1N1) influenza virus pandemic: A review. Vaccine 2010, 28 (31), 4895–4902. .**
6. **J. Kaner, S. Schaack. Understanding Ebola: the 2014 epidemic. Global. Health 2016, 12 (1), 53. .**
7. **Wu P, Hao X, Lau EH, Wong JY, Leung KS, Wu JT, Cowling BJ, Leung GM: Real-time tentative assessment of the epidemiological characteristics of novel coronavirus infections in Wuhan, China, as at 22 January 2020. *Eurosurveillance* 2020, 25(3):2000044**
8. **Thompson R: Pandemic potential of 2019-nCoV. The Lancet Infectious Diseases 2020, 20(3):280**.
9. Team EE: **Note from the editors: World Health Organization declares novel coronavirus (2019-nCoV) sixth public health emergency of international concern**. *Eurosurveillance* 2020, **25**(5).
10. Uddin MN, Alam B, Islam SS, Arif M, Alam MM, Kabir SL: **Impact of COVID-19 on food safety and security in low and middle income countries**. *Asian Journal of Medical and Biological Research* 2020, **6**(2):130-137.
11. **Organization WH: Considerations in adjusting public health and social measures in the context ofCOVID-19: interim guidance, 16 April 2020. In.: World Health Organization; 2020.**
12. Gupta L, Agarwal V, Davalbhakta S, Agarwal V, Misra D: **A survey-based study on the knowledge, attitude, and the practices pertaining to the 2019 novel Corona Virus infection amongst undergraduate medical students in India**. *medRxiv* 2020.
13. Peng Y, Pei C, Zheng Y, Wang J, Zhang K, Zheng Z, Zhu P: **Knowledge, attitude and practice associated with COVID-19 among university students: a cross-sectional survey in China**. 2020.
14. Gallè F, Sabella EA, Da Molin G, De Giglio O, Caggiano G, Di Onofrio V, Ferracuti S, Montagna MT, Liguori G, Orsi GB: **Understanding Knowledge and Behaviors Related to CoViD–19 Epidemic in Italian Undergraduate Students: The EPICO Study**. *International Journal of Environmental Research and Public Health* 2020, **17**(10):3481.
15. Olaimat AN, Aolymat I, Shahbaz HM, Holley RA: **Knowledge and Information Sources About COVID-19 Among University Students in Jordan: A Cross-Sectional Study**. *Frontiers in Public Health* 2020, **8**:254.
16. Baig M, Jameel T, Alzahrani SH, Mirza AA, Gazzaz ZJ, Ahmad T, Baig F, Almurashi SH: **Predictors of misconceptions, knowledge, attitudes, and practices of COVID-19 pandemic among a sample of Saudi population and its impact: a cross-sectional study**. *medRxiv* 2020.
17. Naser AY, Dahmash EZ, Alwafi H, Alsairafi ZK, Al Rajeh AM, Alhartani YJ, Turkistani FM, Alyami HS: **Knowledge and practices towards COVID-19 during its outbreak: a multinational cross-sectional study**. *medRxiv* 2020.
18. Tomar BS, Singh P, Nathiya D, Suman S, Raj P, Tripathi S, Chauhan DS: **Indian communitys Knowledge, Attitude & Practice towards COVID-19**. *medRxiv* 2020.

19. Rugarabamu S, Ibrahim M, Byanaku A: **Knowledge, attitudes, and practices (KAP) towards COVID-19: A quick online cross-sectional survey among Tanzanian residents**.
20. Ssebuufu R, Sikakulya F, Binezero SM, Wasingya L, Nganza SK, Ibrahim B, Kyamanywa P: **Awareness, knowledge, attitude and practice towards measures for prevention of the spread of COVID-19 in the Ugandans: A nationwide online cross-sectional Survey**. *medRxiv* 2020.
21. Al-Hazmi A, Gosadi I, Somily A, Alsubaie S, Saeed AB: **Knowledge, attitude and practice of secondary schools and university students toward Middle East Respiratory Syndrome epidemic in Saudi Arabia: A cross-sectional study**. *Saudi journal of biological sciences* 2018, **25**(3):572-577.
22. Suresh K, Chandrashekara S: **Sample size estimation and power analysis for clinical research studies**. *Journal of human reproductive sciences* 2012, **5**(1):7.
23. Bhagavathula AS, Aldhaleei WA, Rahmani J, Mahabadi MA, Bandari DK: **Novel Coronavirus (COVID-19) Knowledge and Perceptions: A Survey on Healthcare workers**. *medRxiv* 2020.
24. Zhong B-L, Luo W, Li H-M, Zhang Q-Q, Liu X-G, Li W-T, Li Y: **Knowledge, attitudes, and practices towards COVID-19 among Chinese residents during the rapid rise period of the COVID-19 outbreak: a quick online cross-sectional survey**. *Int J Biol Sci* 2020, **16**(10):1745-1752.
25. orld health organization. Emerging respiratory viruses iC-m.
26. Taber KS: **The use of Cronbach's alpha when developing and reporting research instruments in science education**. *Research in Science Education* 2018, **48**(6):1273-1296.
27. Maheshwari S, Gupta PK, Sinha R, Rawat P: **Knowledge, attitude, and practice towards coronavirus disease 2019 (COVID-19) among medical students: A cross-sectional study**. *Journal of Acute Disease* 2020, **9**(3):100.
28. StataCorp L: **Stata statistical software (version release 14)**. *College Station, TX: Author* 2015.
29. **Al Ahdab S: Knowledge, Attitudes and Practices (KAP) towards pandemic COVID-19 among Syrians. 2020.**
30. **Saqlain M, Ahmed A, Gulzar A, Naz S, Munir MM, Ahmed Z, Kamran S: Public Knowledge and Practices regarding COVID-19: A cross-sectional survey from Pakistan. medRxiv 2020.**
31. **Mohamed A, Elhassan E, Mohamed AO, Mohammed AA, Mahgoop MA, Sharif ME, Bashir MI, Abderlahim RB, Idriss WI, Malik EM: Knowledge, attitude and practice of the Sudanese people towards COVID-19: An online survey. 2020.**
32. **Saefi M, Fauzi A, Kristiana E, Adi WC, Muchson M, et al. Survey data of COVID-19-related Knowledge, Attitude, and Practices among Indonesian Undergraduate Students. Data in Brief 2020:105855.**
33. **Paudel S, Shrestha P, Karmacharya I, Pathak OK. Knowledge, attitude, and practices (KAP) towards COVID-19 among Nepalese residents during the COVID-19 outbreak: An online crosssectional study. 2020.**
34. Tadesse AW, Melese N, Eshetie S, Chane M, Ali A: **Knowledge, Attitude, and Practice and Associated Factors towards COVID-19 among College Students in Amhara Region, Ethiopia; A Cross-Sectional Study**. 2020.

Dear Academic Editor!

PLOSE ONE

Response to Academic Editor

I am pleased to resubmit revision (R4) for publication version of "**Assessment of undergraduate student knowledge, practices, and attitude towards COVID-19 in Debre Berhan University**, Ethiopia for a review as original research in **Plose one .**The comments of the editor were highly insightful and enabled us to greatly improve the quality of our manuscript. Therefore, based on the editor's concerns we have made extensive edition in our manuscript. Especially we have extensively edited the manuscript again for language usage, spelling, and grammar before submitting the revised version. The formatting of the text and document (text sizes and grammatical errors) were also edited. In the following pages, we have addressed yours' concerns in a point by point format.

We look forward to hearing from you at your earliest convenience.

Thank you for your consideration of this manuscript!

Kind regards,

Yared Asmare Aynalem.

On behalf of authors

PONE-D-20-14158R3

Assessment of undergraduate student knowledge, attitude, and practices, towards COVID-19 in Debre Berhan University, Ethiopia

PLOS ONE

Dear Dr. Aynalem,

Thank you for submitting your manuscript to PLOS ONE. After careful consideration, we feel that it has merit but does not fully meet PLOS ONE's publication criteria as it currently stands. Therefore, we invite you to submit a revised version of the manuscript that addresses the points raised during the review process.

Response: Thank you very much for allowing us to revise our manuscript again. We have tried to response each concerns as much as possible. We have submitted a revised version of the manuscript

I thank you for the revisions that you have conducted, but I would urge you once more to carefully review the manuscript in terms of clarity and grammar. This is not the usual request for authors to ensure that the English language in the manuscript is adequate, but to ensure that the manuscript is readable and understandable. The paper has had several rounds of reviews and opportunities for these issues to be resolved, so I would be grateful if you could ensure they are resolved. It is disconcerting to find so many issues despite assurances that they have been resolved.

Response: Thank you for allowing us to edit it again. The comments of the editor and the reviewers were highly insightful and enabled us to greatly improve the quality of our manuscript. Therefore, based on your concerns we have made extensive edition in our manuscript. Your comments were highly insightful and enabled us to greatly improve the quality of our manuscript. Therefore, based on the editor's concerns we have made extensive edition in our manuscript. Especially

we have extensively edited the manuscript again for language clarity thoroughly edited the manuscript for language usage, spelling, and grammar before submitting the revised version. The formatting of the text and document (text sizes and grammatical errors) were also edited.

In addition to the comments and recommendations mentioned by the reviewers, I have read the manuscript again and I would like the authors to resolve these pending matters:

Response: Dear our respected editor thank you very much for raising the following issues

Background

2nd para, why is the mortality compared with the flu?

Response: Dear our respected editor thanks you very much. The reason for comparing COVID-19 with the flu is that to show the proportion of mortality as a new outbreak. At the time of seasonal flue outbreak the mortality rate were almost 1%.more over covid mortality rate is almost more than 7 percent .This death rate is particularly alarming. If you are still not comfortable with this sentence, we will remove it.

End of 2nd para, and start of 3rd para, the data does not match- please review and clarify.

Response: Thanks you very much our respected editor .We have rewrite it again; please see the color change from the main document

Ethics statement

Review wording- "ethical approval commute" should be committee - please review across the manuscript. Review the paragraph for clarity.

Response: Thanks you very much our respected editor. We have copy edited it. see also the color change from the ma document

Response: Thank you dear editor. We have clearly descried the contribution on the on the online author contribution form.

Background

2nd para, says "2215 countries", must be a typo.

Response: thanks our beloved editor We have rewritten it. Please See the color change

Reference in pg 16 [9,10] appears in cursive font, please edit.

Response: Thanks .We has corrected it. Please see the color change

End of page: "study conducted in different parts of their world" - say "international" or say which parts of the world.

Response: Thanks .We tried to rephrase & replace with your suggestive appropriate word so called "international"

Methods

Please remove "Abve sea level and temp" unless relevant to the study. Please edit "Collage of ..." To "College of..."

Response: Thanks .We has corrected it. See the color change

Justify socio-demographic variables, marital status, religion - why collected?

Response: we would like to thanks our editor for raising these important concerns. The rational for collecting socio-demographic variables, marital status, and religion is as follows

1. In our country context, the distribution of COVID_19 is high among aged individual & most of adult generation perceived that they are less risky of contracting with it. For that they didn’t want to get even information on it. Therefore, we have collected it as a factor.

2. Most of the community including students talk as COVID is a sin of us.it is from alah,God. So that, not practicing a percussion. This document and risk assessment tool provides practical guidance and recommendations to support the special role of religious leaders, faith-based organizations, and faith communities in COVID-19 education, preparedness, and response.
3. Additionally, differ study conducted globally, shows that socio-demographic variables, marital status, religion are linked with COVID-19 KAP. Here, we examine how KAP varies across fundamental socio-demographic characteristics, including age, sex, civil status, individual disposable income, religion of. We have collected it to for identifying the gap & to intervene on it .

Was the data collection validated?

Was tool validated in students or only faculty?

"Ensure genuine replies..." - how did they do that?

Thanks dear reviewer..!!! The proposed tool was developed and validated by a multidisciplinary working group of infectious disease physicians, lecturers (Public health, nurse & environmental health professionals, and infectious diseases Public health professionals. The working group regularly reviewed literature to select important characteristics and outcomes for inclusion. We also used a

The development phase was conducted at Debre birhan University health science collage and the pilot validation phase was conducted at the nursing department students. We have also done a pretest from 5 % of the student.   The team members formed a working group that met via telephone or video conferences at least biweekly

Ethics consideration

Commute = committee,Paper says participation anonymous but later on it mentioned that collects personal details like phone? Please clarify

Response: Thanks .We has corrected it. See the color change

Results

What was the total number of participants approached, to give the 86.1% rate? 634

Response: Thanks dear editor.as we have stated in sample size determination, we have included approached to a total of 634 participate.

Discussion

End of 1st para: please add evidence that "Students get regular update through the medias"

Response: Dear editor thanks for raising the concern. We have given a possible justifications based o our experience of giving the student about the outbreak COVID-19. We have given to them a health education program for all faculty of the university before the school was closed. Possibly this may increase their knowledge

2nd and 3rd para need punctuation mark at the end.

Response : Dear editor thanks for raising the concern. We have tried to edit all parts of the paper.

Response: thank you for your critical review dear editor. We have corrected it .See also the color change

Please submit your revised manuscript by Jan 24 2021 11:59PM. If you will need more time than this to complete your revisions, please reply to this message or contact the journal office at plosone@plos.org. When you're ready to submit your revision, log on to https://www.editorialmanager.com/pone/ and select the 'Submissions Needing Revision' folder to locate your manuscript file.

Please include the following items when submitting your revised manuscript:

A rebuttal letter that responds to each point raised by the academic editor and reviewer(s). You should upload this letter as a separate file labeled 'Response to Reviewers'.

A marked-up copy of your manuscript that highlights changes made to the original version. You should upload this as a separate file labeled 'Revised Manuscript with Track Changes'.

An unmarked version of your revised paper without tracked changes. You should upload this as a separate file labeled 'Manuscript'.

Response: Thank you. We have included ‘Response to Reviewers', 'Revised Manuscript with Track Changes & Manuscript separately

If you would like to make changes to your financial disclosure, please include your updated statement in your cover letter. Guidelines for resubmitting your figure files are available below the reviewer comments at the Editorial manager (https://www.editorialmanager.com/pone/)

Response: Thank you. We dot like to change the financial disclosure

We look forward to receiving your revised manuscript.

Kind regards,

Enrique Castro-Sánchez

Academic Editor

PLOS ONE

Reviewers' comments:

Reviewer's Responses to Questions

Comments to the Author

1. If the authors have adequately addressed your comments raised in a previous round of review and you feel that this manuscript is now acceptable for publication, you may indicate that here to

bypass the “Comments to the Author” section, enter your conflict of interest statement in the “Confidential to Editor” section, and submit your "Accept" recommendation.

Reviewer #3: All comments have been addressed

Response: dear reviewer, we thank you for spending your valuable time to improve this paper. We have a grate appreciation for helping us

2. Is the manuscript technically sound, and do the data support the conclusions?

The manuscript must describe a technically sound piece of scientific research with data that supports the conclusions. Experiments must have been conducted rigorously, with appropriate controls, replication, and sample sizes. The conclusions must be drawn appropriately based on the data presented.

Reviewer #3: Yes

Response: Thank you dear reviewer

3. Has the statistical analysis been performed appropriately and rigorously?

Reviewer #3: Yes

Response: Thank you dear reviewer

4. Have the authors made all data underlying the findings in their manuscript fully available?

The PLOS Data policy requires authors to make all data underlying the findings described in their manuscript fully available without restriction, with rare exception (please refer to the Data Availability Statement in the manuscript PDF file). The data should be provided as part of the manuscript or its supporting information, or deposited to a public repository. For example, in addition to summary statistics, the data points behind means, medians and variance measures should be available. If there are restrictions on publicly sharing data—e.g. participant privacy or use of data from a third party—those must be specified.

Reviewer #3: Yes

Response: Thank you dear reviewer

5. Is the manuscript presented in an intelligible fashion and written in standard English?

PLOS ONE does not copyedit accepted manuscripts, so the language in submitted articles must be clear, correct, and unambiguous. Any typographical or grammatical errors should be corrected at revision, so please note any specific errors here.

Reviewer #3: Yes

Response: Thank you dear reviewer

6. Review Comments to the Author

Please use the space provided to explain your answers to the questions above. You may also include additional comments for the author, including concerns about dual publication, research ethics, or publication ethics. (Please upload your review as an attachment if it exceeds 20,000 characters)

Reviewer #3: Authors have adequately addressed my comments in this new version of the article. Discussion section provides more concrete information.

Response: Thank you dear reviewer. It is your effort too

7. PLOS authors have the option to publish the peer review history of their article (what does this mean?). If published, this will include your full peer review and any attached files.

If you choose “no”, your identity will remain anonymous but your review may still be made public.

Do you want your identity to be public for this peer review? For information about this choice, including consent withdrawal, please see our Privacy Policy.

Reviewer #3: Yes: Ramon Flecha

Response: Thank you dear reviewer

Dear all (both the reviewer ad editors),we would like to appreciate all your effort for your effort to improve our paper .may God paid all your effort. We have get differ research skills from your point of view

Thanks
