# Supplementary material for: Assessment of undergraduate student knowledge, attitude, and practices towards COVID-19 in Debre Berhan University, Ethiopia
Source: PLoS One. 2021 May 18;16(5):e0250444. doi: 10.1371/journal.pone.0250444 (PMC8130923; doi:10.1371/journal.pone.0250444)
Supplement: Attachment — Submitted filename: Response to Reviewers.docx [file pone.0250444.s003.docx]

**Dear Academic Editor!**

**PLOS ONE**

**Response to Academic Editor and Reviewers**

I am pleased to resubmit for publication a revision version of “**Assessment of undergraduate student knowledge, practices, and attitude towards COVID-19 in Debre Berhan University, Ethiopia**” for a review as original research in **PLOS ONE**

.The comments of the editor and the reviewers were highly insightful and enabled us to greatly improve the quality of our manuscript. Therefore, based on the editor’s and the reviewers’ concerns we have made extensive edition in our manuscript. The comments of the editor and the reviewers were highly insightful and enabled us to greatly improve the quality of our manuscript. Therefore, based on the editor’s and the reviewers’ concerns we have made extensive edition in our manuscript. Especially we have extensively edited the manuscript by a professional language editor, at **Excision Editing** (a fluent, native Australian, English-language speaker thoroughly edited the manuscript for language usage, spelling, and grammar) before submitting the revised version. The formatting of the text and document (text sizes and grammatical errors) were also edited. His name is **called Dr. Ryan Bell (CEO and Chief Editor Excision Editing)**

In the following pages, we have addressed yours’ concerns in a point by point format.

We look forward to hearing from you at your earliest convenience.

Thank you for your consideration of this manuscript!

Kind regards,

**Yared Asmare Aynalem.**

On behalf of authors

PLOS ONE Decision: Revision required [PONE-D-20-14158] - [EMID:3076548e22979785]

| Inbox | x |
| --- | --- |

**PLOS ONE**

PONE-D-20-14158
Assessment of undergraduate student knowledge, practices, and attitude towards COVID-19 in Debre Berhan University, Ethiopia
PLOS ONE

Dear Dr. Aynalem,

Thank you for submitting your manuscript to PLOS ONE. After careful consideration, we feel that it has merit but does not fully meet PLOS ONE’s publication criteria as it currently stands. Therefore, we invite you to submit a revised version of the manuscript that addresses the points raised during the review process.

Response: Thank you very much for allowing us to revise our manuscript. We have tried to response each concerns as much as possible. We have submitted a revised version of the manuscript.

The reviewers have appreciated the urgency required to generate evidence regarding an emerging global pandemic such as covid-19, and praise in particular the benefit of such data from African settings. However, they have raised key methodological concerns and in particular, I would urge to consider the reviewers' comments, concerns and suggestions regarding the methodological aspects of the study, and the required improvement in the reporting of the conduct (sampling, selection, etc) of the research.  I would also like you to appraise the comments offered by Reviewer 4 regarding some of the analytical decisions that you have made in the paper, and ensure that a response to all the points raised by the Reviewer is included.  Finally, I would suggest that attention to the novelty aspects of the paper are not really required, as PLOS ONE focuses on the technical quality of the submitted work. 
Response: Thank you very much for recognizing the urgency required to generate evidence regarding an emerging global pandemic of covid-19, and giving praise to us .really excuse for missing these important methodological issues from the main manuscript. For that, we have included the detail methodological descriptions of the current study. Dear editor please see the color change in the manuscript.
Please submit your revised manuscript by Aug 24 2020 11:59PM. If you will need more time than this to complete your revisions, please reply to this message or contact the journal office at [plosone@plos.org](mailto:plosone@plos.org). When you're ready to submit your revision, log on to <https://www.editorialmanager.com/pone/> and select the 'Submissions Needing Revision' folder to locate your manuscript file.

Response: Thank you. We have submitted per the due date.

Please include the following items when submitting your revised manuscript:

- A rebuttal letter that responds to each point raised by the academic editor and reviewer(s). You should upload this letter as a separate file labeled 'Response to Reviewers'.
- A marked-up copy of your manuscript that highlights changes made to the original version. You should upload this as a separate file labeled 'Revised Manuscript with Track Changes'.
- An unmarked version of your revised paper without tracked changes. You should upload this as a separate file labeled 'Manuscript'.

Response: Thank you. We have included ‘Response to Reviewers', 'Revised Manuscript with Track Changes ad Manuscript separately

If you would like to make changes to your financial disclosure, please include your updated statement in your cover letter. Guidelines for resubmitting your figure files are available below the reviewer comments at the end of this letter.

Response: thanks for allowing us to update the financial disclosure .we have added to the cover later .We didn’t have any figure .

If applicable, we recommend that you deposit your laboratory protocols in [protocols.io](http://protocols.io/) to enhance the reproducibility of your results. Protocols.io assigns your protocol its own identifier (DOI) so that it can be cited independently in the future. For instructions see: <http://journals.plos.org/plosone/s/submission-guidelines#loc-laboratory-protocols>

Response: thank you for recommending depositing the laboratory protocols in protocols.io. But it is not applicable

We look forward to receiving your revised manuscript.

Kind regards,

Enrique Castro-Sánchez
Academic Editor
PLOS ONE.

Response: Thank you .we has set it
Journal Requirements:

When submitting your revision, we need you to address these additional requirements.

p1. Please ensure that your manuscript meets PLOS ONE's style requirements, inc;luding those for file naming. The PLOS ONE style templates can be found at <https://journals.plos.org/plosone/s/file?id=wjVg/PLOSOne_formatting_sample_main_body.pdf> and <https://journals.plos.org/plosone/s/file?id=ba62/PLOSOne_formatting_sample_title_authors_affiliations.pdf>

Response: thank you very much. We have sent the revised manuscript as based on the plose one guideline and as per editor’s comment

2.  In the Methods, please clarify that participants provided oral consent. Please also state in the Methods:

- Why written consent could not be obtained, Whether the Institutional Review Board (IRB) approved use of oral consent, How oral consent was documented.For more information, please see our guidelines for human subjects research: <https://journals.plos.org/plosone/s/submission-guidelines#loc-human-subjects-research>"

**Response:** Dear our editor, we would like to say very excuse for making you confused .it was as an error. We have taken written informed consent. It was stated as Ethical clearance for this study was obtained from the Institute of Medicine and College of Health Sciences, DBU. **It was approved by the University** ethical approval commute with the approval number of Ref. **No.DBU.R.D136/02/12.** Participants also gave a written informed consent prior to data collection after the participates are informed that their participation is completely voluntary and anonymous, if they decide to take part, still free to withdraw at any time, all information that is collected will kept strictly confidential, personal details such as phone number and address will not be revealed to people outside the research teams. Confidentiality of the study participants' information was maintained throughout the study by making the participants' information anonymous. (see also tools that we used ad attached as the supplmetary files).

3. Please include additional information regarding the survey or questionnaire used in the study and ensure that you have provided sufficient details that others could replicate the analyses. For instance, if you developed a questionnaire as part of this study and it is not under a copyright more restrictive than CC-BY, please include a copy, in both the original language and English, as Supporting Information."

Response: thanks for allowing us to add it .We have attached both the Amharic and English version of questionnaire used in the study (see also tools that we used ad attached as the supplmetary files).
4. We noticed minor instances of text overlap with the following previous publication(s), which need to be addressed:

(a) <https://www.ijbs.com/v16p1745.pdf>

(b) <https://news.mb.com.ph/2020/02/06/who-crafts-global-strategic-plan-vs-ncov/>

(c) <https://en.wikipedia.org/wiki/Pandemic>.The text that needs to be addressed involves the Introduction section.In your revision please ensure you cite all your sources, and quote or rephrase any duplicated text outside the methods section. Further consideration is dependent on these concerns being addressed."

Response: thanks for your recommendation for editing a text overlap o the above papers. The comments of the editor were highly insightful and enabled us to greatly improve the quality of our manuscript. Therefore, based on the editor’s and the reviewers’ concerns we have made extensive edition in our manuscript. Especially we have extensively edited the manuscript by a professional language editor, at Excision Editing (a fluent, native Australian, English-language speaker thoroughly edited the manuscript for language usage, spelling, and grammar) before submitting the revised version. The formatting of the text and document (text sizes and grammatical errors) were also edited. We have also tried to cite all sources.

5. In your Methods section, please provide additional information about the participant recruitment method and the demographic details of your participants. Please ensure you have provided sufficient details to replicate the analyses such as:  a) a table of relevant demographic details, d) a statement as to whether your sample can be considered representative of a larger population and c) a description of how participants were recruited."

Response: big thanks dear our editor. We have included a table of relevant demographic details as a supplementary files stated as follows. We also included about sample representatives ad a description of how participants were recruited. See the color change

**A table of relevant demographic details,**

| Sr o | Collage | Departments | Total under graduated student in each collage | Included collage data collection(randomly selected) |
| --- | --- | --- | --- | --- |
| 1 | ****School of Computing Science**** | - Information technology - Information system - Computer science - Software Engineering | ****1231**** |  |
| 2 | ****College of Engineering**** | - Electrical  and Computer Engineering - Mechanical engineering - Civil engineering - Chemical engineering - Construction technology and management - Industrial Engineering - Survey Engineering - Food Processing  Engineering | 4286 |  |
| 3 | ****College of Health Science**** | - Nursing - Midwifery - Health officer - Pediatrics Nursing - Neonatal Nursing - Surgical Nursing - Medical Laboratory Science | 616 |  |
| 4 | ****College of**** ****Medicine**** | - Anesthesia - Medicine - Pharmacy | 263 |  |
| 5 | ****College of Natural and Computational Science**** | - Biology - Chemistry - Physics - Mathematics - Sport science - Statistics - Biotechnology - Geology | 1231 |  |
| 6 | ****College of Business and Economics**** | - Management - Economics - Accounting and Finance - Tourism Management - Logistics and Supply Chain Management - Marketing Management | 1257 |  |
| 7 | ****College of Agriculture and Natural Resource Science**** | - Plant science - Animal science - Natural resource management - Water resource and irrigation management - Horticulture - Agricultural Economics | 741 |  |
| 8 | ****College of Social Science and Humanities**** | - Geography and Environmental Studies - History and heritage management - Sociology - Psychology - English language and literature - Amharic - Civics and ethical education - Journalism and Communication | 1210 |  |
| 9 | **College of Law** | Law | 221 |  |
| 10 | **College of Education** | - Technical Drawing  /Summer/ - Business Education /Summer/ - Special Need Education / Summer/ | 1235 |  |

6. Please provide a sample size and power calculation in the Methods, or discuss the reasons for not performing one before study initiation.

Response: Dear editor again we are sorry for the inconvenience. We have added Sample size determination and sampling procedure (procurement procedures) as follows: **Sample size determination and sampling procedure (procurement procedures**)

The sample size was calculated by using single population proportion formula assuming: 95% confidence level, 3% margin of error, and, 50% proportion for KAP level. Since there is no any study in our countries, we have used 50% proportion. After a 10% non-response rate and 1.5 design effects, the final sample size was 634. Our study sample was obtained by a two-stage cluster sampling technique. In the first stage, out of ten collages, four of them (collage of engendering, computing science, business and economics and collage of agriculture) were selected by simple random sampling and sample size was allocated proportionally to get the required sample size from each selected colleges. The sampling fraction is approximately equal to seven for all colleges. In the second stage, the study participants were selected from each year and sections of the selected collage using a systematic random sampling technique every 7^th^ students from each college registrar office log-book. . After that a dorm to dorm visit were used to get the sampled students. Since we have used 50% proportion, our sample can be considered as a representative of a larger population. Students in each selected collage that were present during the data collection period and were able to give responses were included in the study. Those who had mental or physical disabilities and could not fill out the questionnaires (unwilling to participate in to the study) were excluded from the study.

7. Please amend your list of authors on the manuscript to ensure that each author is linked to an affiliation. Authors’ affiliations should reflect the institution where the work was done (if authors moved subsequently, you can also list the new affiliation stating “current affiliation:….” as necessary).

Response: thanks for your recommendation for amending the list of authors on the manuscript. We have amended it ad we have ensured that that each author is linked to an affiliation.

Yared Asmare Aynalem^1*^ Tadess Yirga Akalu^2¶^,, Birhan Gebresellassie^1&^, Nigussie Tadesse Sharew^1& ,^Wondimeneh Shibabaw Shiferaw^1¶^

^1^Department of Nursing, College of Health Science, Debre Berhan University, Debre Berhan, Ethiopia

^2^Department of Nursing, College of Health Science, Debre Markos University, Debre Markos, Ethiopia

^*^Corresponding author: [yaredasmare123@gmail.com](mailto:yaredasmare123@gmail.com)

¶ these authors contributed equally to this work.

& these authors also contributed equally to this work.

8. Please include a copy of Tables 1-5 which you refer to in your text.

Response: thanks for reminding to add the missed Tables 1-5.We have included them. See the color change
Reviewers' comments:

Reviewer's Responses to Questions

**Comments to the Author**

1. Is the manuscript technically sound, and do the data support the conclusions?

The manuscript must describe a technically sound piece of scientific research with data that supports the conclusions. Experiments must have been conducted rigorously, with appropriate controls, replication, and sample sizes. The conclusions must be drawn appropriately based on the data presented.

Reviewer #1: Yes

Reviewer #2: Yes

Reviewer #3: Yes

Reviewer #4: No

Reviewer #5: Partly

Response: thank you very much.I think we have tried to modified it see the track change

2. Has the statistical analysis been performed appropriately and rigorously?

Reviewer #1: I Don't Know

Reviewer #2: Yes

Reviewer #3: Yes

Reviewer #4: No

Reviewer #5: Yes

Response: thank you very much. We have try to revised it

3. Have the authors made all data underlying the findings in their manuscript fully available?

The [PLOS Data policy](http://www.plosone.org/static/policies.action#sharing) requires authors to make all data underlying the findings described in their manuscript fully available without restriction, with rare exception (please refer to the Data Availability Statement in the manuscript PDF file). The data should be provided as part of the manuscript or its supporting information, or deposited to a public repository. For example, in addition to summary statistics, the data points behind means, medians and variance measures should be available. If there are restrictions on publicly sharing data—e.g. participant privacy or use of data from a third party—those must be specified.

Reviewer #1: Yes

Reviewer #2: Yes

Reviewer #3: Yes

Reviewer #4: Yes

Reviewer #5: No

Response: thank you very much. We have included the remaining tool (questioner ) as a supplementary files

4. Is the manuscript presented in an intelligible fashion and written in standard English?

PLOS ONE does not copyedit accepted manuscripts, so the language in submitted articles must be clear, correct, and unambiguous. Any typographical or grammatical errors should be corrected at revision, so please note any specific errors here.

Reviewer #1: Yes

Reviewer #2: No

Reviewer #3: Yes

Reviewer #4: Yes

Reviewer #5: Yes

Response: thank you very much. The comments of the reviewer were highly insightful and enabled us to greatly improve the quality of our manuscript. Therefore, based on the editor’s and the reviewers’ concerns we have made extensive edition in our manuscript. Especially we have extensively edited the manuscript by a professional language editor, at **Excision Editing** (a fluent, native Australian, English-language speaker thoroughly edited the manuscript for language usage, spelling, and grammar) before submitting the revised version. The formatting of the text and document (text sizes and grammatical errors) were also edited. We have also tried to cite all sources.

**5. Review Comments to the Author**

Please use the space provided to explain your answers to the questions above. You may also include additional comments for the author, including concerns about dual publication, research ethics, or publication ethics. (Please upload your review as an attachment if it exceeds 20,000 characters)

Response: thank you very much. We have tried to answer the reviewer’s feedback ad question as much as we can.

**Reviewer 1**
Assessment of undergraduate student knowledge, practices, and attitude towards COVID-19 in Debre Berhan University, Ethiopia.Many thanks for the opportunity to review this paper.
There is a gap for the attitude to the pandemic, especially in Africa on the one hand, but also a flood of publications without a whole lot of purpose. I am slightly undecided whether this article enhances knowledge. Inclined to think to be publishable it needs a follow up element perhaps now or in 6 months time?

Response: thank you very much for reviewing the paper. As far as we know, still there is scarcity of data particularly in low income countries including Ethiopia. This data may also help for the future systematic review ad meta-analysis to synthesis results.

Abstract
I feel like the abstract doesn’t really give us key information on the findings – all I get from it is that some were less knowledgeable and there is a concern about educating them. Is that it? Hoping more specific as I read article.

Response: We thank you very much for allowing us to improve the abstract of this finding. We have tried to improve it. See the color change please.
Background
Some of the data is now 2 months old and outdated.. but I guess you had to draw line somewhere. Repeated this line twice “seasonal flu generally kills far fewer than 1% of those infected[9]” The Chinese study [10] was it before/ during/ after the surge in China as to whether comparable to your data (before). I’m not sure quoting all the WHO strategies is helpful/ necessary. The whole 2nd last paragraph of the background is very much public knowledge at this stage and I don’t believe adds much.

Response: We thank you very much for the concern. We have used the latest /updated report of data as per your constructive feedback .thank you for that. We have also removed the repeated sentence. We have also modified the WHO strategies which is helpful to compate COVID-19 currently

**Methods.**
What is the intended faculty/ field of study of the students?? Questionnaire seems appropriate and rigorous.

Response: Thank you. The source population of this study was all undergraduate students at Debre Berhan University	and the study population was all students in the randomly selected collages (Collage of engendering, computing science, Faculty of business and economics	and collage of agriculture).

Results
The field of study of the students?? I think this is vital. I would expect science/ health students to know more? Far more interested in this than their gender and age myself.
Response: since we have selected 4 collages randomly (see the sampling procedure) (Collage of engendering, computing science, Faculty of business and economics and collage of agriculture) we didn’t report each separately. Because the finding of this study is generalized to all collages. Additionally we have attached a supplemtry files that aides the population

No Table 1, 2 in the version I have been given to view.
The results are succinct. Fine. I guess interesting in retrospect in years to come especially. But not remarkable – mostly as I would expect from educated students at this stage of the pandemic.
Response: thanks for reminding to add the missed Tables 1-5.We have included them. See the color change

Discussion
I find this pretty superficial and repeating the results. Not a whole lot of interpretation. How does this compare even to the Chinese study you mentioned??You don’t really express an opinion even..
Response: thanks .We have rewrite the overall parts of the discussion by comparing with differ finding to the world. Please see the color change

Concl & Recom
Again you don’t really pull out what is novel/ surprising. What about recommendations for repeating this research later?All you give is rather vague “educate” messages. How? Social media seems to work??

Response: thanks for asking us to improve the conclusion and recommendation. We have revised this part intensively. We have tried to make our recommendations as specific as possible See the color change.

Reviewer #2: Covid19 related research is timely and should be supported. The effort of the authors is therefore praise worth.

Response: Thank you for acknowledging that
However
• There are some grammatical errors that should be corrected.

Response: Thank you. The comments of the reviewer were highly insightful and enabled us to greatly improve the quality of our manuscript. Therefore, based on the editor’s and the reviewers’ concerns we have made extensive edition in our manuscript. Especially we have extensively edited the manuscript by a professional language editor, at **Excision Editing** (a fluent, native Australian, English-language speaker thoroughly edited the manuscript for language usage, spelling, and grammar) before submitting the revised version. The formatting of the text and document (text sizes and grammatical errors) were also edited.
• At the discussion section, the authors should compare their findings with what others have written before.

Response: Thank you for your golden feedback. We have discussed it as per your suggestions
• The recommendations on educational campaigns should be made more explicit.

Response: Thank you. We have tried to make our recommendations as specific as possible See the color change.
• The reference section should be checked for consistency, e.g. some emboldens

Response: Thank you. We have revised all part of the reference

Reviewer #3: The paper presents an interesting and relevant research about the association between knowledge, attitude and practices (KAP) of Debre Berhan University students, towards COVID-19. The abstract presents the basic information regarding background, methods, results and conclusions, but the main objective of the paper is missing. To clarify the main aim of the article an objective needs to be included. Considering that the (KAP) towards COVID-19 is a central element, the title of the paper could be modified to adjust to that: “Assessment of undergraduate student knowledge, attitude and practices towards COVID-19 in Debre Berhan University, Ethiopia”.

Response: Thank you for acknowledging that. We have included an objective at the abstract part
The background section provides the main contents regarding COVID-19. The study took place in March and for this reason references about COVID-19 are also from March-April. However, as far as possible, it could be positive to have more recent data regarding confirmed cases, number of deaths and the number of cases reported in Debre Berhan and Ethiopia.

Response: Thank you. We have included the most recent data as per your suggestion. See the color change.

Just a brief comment regarding the information provided in the third paragraph regarding the countries that have been reporting COVID-19 cases. In this explanation the number of countries provided is 2214, but it is not possible, please revise the data.

Response: Thank you. We have included the most recent data as per your suggestion (including worldometer report). See the color change.

At the end of this section a direct link is made between one study in China and the (KAP) towards the disease. It could be relevant to provide more analysis about how this association is made in other studies, particularly because this relation is essential in your study.

Response: Thank you for your golden feedback. We have discussed it as per your suggestions. See the color change.

In the sub-section “study design, setting, and population” concrete and basic information regarding the population and the setting where the field work took place is included. However, little information about the study design is mentioned. Introducing here some explanation showing your design would be relevant for the article.

Response: Thank you for you. A cross-sectional study was conducted from March 18–24, 2020, with undergraduate DBU students. For more detail please also see the color change.

The information regarding the study variables is clear, with the outcome variable and the two independent covariates. But there is no explanation establishing the relation between both types of variables. Establishing a link between both through a research question would be important, because later, a binary logistic regression analysis to select factors has been done.
Data collection tool and procedure is correct in general terms, but the following aspects need to be improved:
Response: Thank you :In this study, knowledge (good/poor), attitude (positive/negative), practice (good/poor) towards COVID-19 was the outcome variable. Socio-demographics factor like age, residence, sex, marital status, educational level, field of study, income, family size, religion) and the source of information regarding COVID-19 were considered the independent covariates. Knowledge for attitude, knowledge and attitude for practice towards the preventive measures of COVID-19 were also assessed. Correlation between independent variables (including the correlation between knowledge, attitude ad practice) was assessed .But we did not find any Correlation

- The explanation about the questionnaire. There are two parts and the first one, based in socio-demographic variables, is clear. But at the beginning of the second one regarding KAP, it would be necessary to explain that there are three different type of questions and a different way to work with the data and after that, provide an explanation of the three of them.

Response: Thank you for your feedback .We have modified it as follows: The questionnaire consisted of four parts: 1) socio-demographics; 2) knowledge; 3) attitudes; and 4) practices of to COVID-19. Socio-demographic variables included age, sex, and marital status, and residence, year of study, average monthly income, and source of information.

- The explanation about the knowledge about COVID-19 is correct, but it is important to justify why you have established three options per question when you are developing later a binary regression analysis. It is true that at the end you work with two - as you classify “No” and “I don’t know” as zero and “Yes” as one- but explaining the reasons why you decide to work in that way would be necessary.

Response: thank you dear reviewer r .due to the small number of participates responding as in don’t know, we have recatagorized.
- In the case of attitudes towards COVID-19 a five-point Likert scale has been used (strongly agree, agree, neutral, disagree, and strongly disagree). However, at the end, and following previous studies, three categories (agree, neutral and disagree) have been established. First of all, it might be useful to specify which are these previous studies? and second, you need to clarify the content of each category. Rationally “strongly agree” and “agree” must be in the category “agree” and “disagree” and “strongly disagree” in the category “disagree”.

Response: thank you dear reviewer. We have cited the previous study that we used as a base line. Strongly agree and agree were category as “agree” and “disagree” and “strongly disagree” in the category of “disagree. The additional rational is due to the small number of participate.

- Information regarding participants’ practices needs more development. Which kind of questions are addressed to assess the participants’ practices? Are there dichotomic or is a Likert scale used? How many questions are included in this case?
- Finally, a short explanation about how the questionnaire is administered could be introduced.
Response: thank you dear reviewer. We have used dichotomic to assess the participants’ practices based on the who tools and different finding that we have cited .See also the color change .

Data processing and analysis section contains different types of statistics’ analysis. In the case of the descriptive statistics a relation between study participants and the “relevant variables” is suggested but insufficient information is provided concerning these relevant variables.
Response: thank you dear reviewer. Sorry for making you confused. We have made a correction (to show variables of Socio-demographic characteristics, participants’ knowledge, and attitude ad practice regarding COVID-19).
In the case of the multivariable analysis, a binary logistic regression analysis to identify the factors associated with KAP is used. Later, the factors were selected using backward stepwise method, but there is no information regarding this process, the main factors selected for the study and how they have been selected.

### Response: thank you dear reviewer .dear reviewer .we have modified it .We revised and reanalysis using the default (Entry Method), which is the standard method of entry is simultaneous (the enter method); all independent variables are entered into the equation at the same time.   Each predictor is assessed as though it were entered after all the other independent variables were entered, and assessed by what it offers to the prediction of the dependent variable that is different from the predictions offered by the other variables entered into the model.

Results are presented by each type of statistics’ analysis, including the main information and being very clear in that way. The discussion section provides complementary explanations to the results.

Response: thank you dear reviewer for acknowledging that

Finally, regarding conclusions and recommendation, including the main objective of the paper at the beginning would be positive and after that, the argumentation can follow. The recommendation about educational campaigns that are included just at the end is too short, more information could be provided on this.
Response: thank you. We have addressed your concern dear reviewer. Please see the color change
A final comment regarding the references: the title or sometimes the authors appear in bold in all of them. Please check all references and make sure that all of them follow the journal’s requirements in this matter.

Response: thank you. We have corrected it .see the main document please

Reviewer #4: 1. The information provided about the data analysis is insufficient. In particular, coding of outcome variables in the regression models is unclear.

Response: we the authors would like to thanks for your valuable feedback. We tried to modified it as sufficient as possible per your suggestion (see the color change).the outcome variables were coded as follows: Coding of outcome variables in the regression models were stated as follows. 1 point was given to adequate, and 0 inadequate in the Knowledge; 1 for positive, and 0 for negative in the Attitude; 1 for good, and 0 for poor in the practice part. See also the color change
2. The authors do not provide reasons for using multivariate analysis with binary dependent variable.

Response: Very excuse dear reviewer it is a typing error. It is to mean Multivariable regression results of the binary logistic regression analyses that control cofounding, not to mean multivariate analysis.

3. The stepwise method is widely criticized in the literature. I do not find it appropriate either.

Response: dear reviewer we acknowledge your feedback

### Certain regression selection approaches are helpful in testing predictors, thereby increasing the efficiency of analysis. Therefore, we thanks for allowing us to select the best regression selection approaches. We revised and reanalysis using the default (Entry Method), which is the standard method of entry is simultaneous ( the enter method); all independent variables are entered into the equation at the same time.   Each predictor is assessed as though it were entered after all the other independent variables were entered, and assessed by what it offers to the prediction of the dependent variable that is different from the predictions offered by the other variables entered into the model.

4. The strict use of 5% limit for discussing significance is not in line with current consensus in the literature of statistical analysis.

Response: dear reviewer thank you for your up to dated evidence of it. **b**ut we didn’t got any new evidence that supports to declare significance .We have tried to read more including evidences entitled Moving to a World Beyond “p < 0.05” y Ronald L. Wasserstein, Allen L. Schirm & Nicole A. Lazar. Still they suggested importantly, when reporting p values, authors should always provide the actual value, not only statements of “p < 0.05” or “p ≥ 0.05”, because p values give a measure of the degree of data compatibility with the null hypothesis. Please suggest if any

Reviewer #5: Thanks for the opportunity to revise this paper.

In this research, the authors present research on the knowledge, practices, and attitude towards COVID-19 amongst undergraduate students in a university of Ethiopia.

My major concern with the article relates to the explanation of the sample obtained. There is a void of information at that stage, while the remaining, methods, variables, analysis, are correctly reported. In particular, no description at all is provided on how the sample was selected. What population does the sample represent? if any. As it is described, the sample seems to be a convenient sample (people ‘around’ were selected), not a representative sample of those 11000 undergraduate students. What were the response rates? The authors mentioned exclusions, but who were they and what were the reasons. Did the authors conduct any power calculation? The authors claimed that the sample was of limited size, but it was above 500. What is large or small depends on the purpose of the sample, but this remains unclear without a power calculation**.** Why the authors reported to be of limited size remains unclear.

Response: thank you. Dear reviewer sorry for the inconvenience. We have made extensive edition on this issue. The sampling methods are modified as follows:

The sample size was calculated by using single population proportion formula assuming: 95% confidence level, 3% margin of error, and, 50% proportion for KAP level. Since there is no any study in our countries, we have used 50% proportion. After a 10% non-response rate and 1.5 design effects, the final sample size was 634. Our study sample was obtained by a two-stage cluster sampling technique. In the first stage, out of ten collages, four of them (collage of engendering, computing science, business and economics and collage of agriculture) were selected by simple random sampling and sample size was allocated proportionally to get the required sample size from each selected colleges. The sampling fraction is approximately equal to seven for all colleges. In the second stage, the study participants were selected from each year and sections of the selected collage using a systematic random sampling technique every 7^th^ students from each college registrar office log-book. . After that a dorm to dorm visit were used to get the sampled students. Since we have used 50% proportion, our sample can be considered as a representative of a larger population. Students in each selected collage that were present during the data collection period and were able to give responses were included in the study. Those who had mental or physical disabilities and could not fill out the questionnaires (unwilling to participate in to the study) were excluded from the study. we has corrected all the mistakes. See the color change
What is missing is a good literature review and discussion of the state of the art in KAP against COVID19 in countries across the world, amongst different groups (health professionals, politicians, general population, young educated people [this study]) across countries and maybe within Ethiopia. The literature used by the authors remains thin, with a lot of focus and quite lengthy description of general advice by WHO. We should know why KAP is relevant in relation to other crises and what are the KAP levels in Ethiopia and other countries. I acknowledge that there is possibly many more papers published now, relative to the time of writing this paper. However, literature for other infectious disease crisis exist, such as for Ebola in Western Africa, and could be helpful to give more depth to this work. Note that this has implications for the Discussion, too.

Response: thank you for your feedback. We have extensively edited the entire paper including the introduction and Discussion **b**y including the latest data countries across the world. Please see the color change

The discussion is clearly too short, was quickly put together, but more importantly, no links are made to any other literature. Some results such as a lower awareness in students from rural areas or an age effect should be discussed in light of other literature out there.

Response: thank you. We have addressed this issue dear reviewer. We try to link our finding from differ parts of the world ad we try to give possible justification as far as we can See the color change

Below I provide a few minor comments,
Abstract, does not include essential methodological features, such as the sample.

Response: thank you. We have addressed this issue. See the change
[Page 1, Introduction] ‘By comparison, seasonal flu generally kills far fewer than 1% of those infected[9]’ This is repeated in the paragraph above. Please avoid repetition
[Page 1, Introduction] ‘A study in China showed that most respondents were knowledgeable about COVID-19 and the vast majority of the participants also held an optimistic attitude towards the COVID-19 pandemic. Specifically, 90.8% believed that COVID-19 would be successfully controlled, and 97.1% had confidence that China could win the battle against the virus [10].’ Add more studies.
References 1, 4, 9 and 15 are incomplete or have typos. Please revise.
Tables are not attached to the submission, so not evaluated.

Response: big thanks. We revised the reference and we think that we have modified. Additionally, we have deleted any redundancy and we have tried to use the latest data .thank you for your golden feedback

6. PLOS authors have the option to publish the peer review history of their article ([what does this mean?](https://journals.plos.org/plosone/s/editorial-and-peer-review-process#loc-peer-review-history)). If published, this will include your full peer review and any attached files.


If you choose “no”, your identity will remain anonymous but your review may still be made public.


**Do you want your identity to be public for this peer review?** For information about this choice, including consent withdrawal, please see our [Privacy Policy](https://www.plos.org/privacy-policy).

Reviewer #1: **Yes:** PW Hodkinson

Reviewer #2: **Yes:** Dr John Bosco Azigwe

Reviewer #3: **Yes:** Ramon Flecha

Reviewer #4: No

Reviewer #5: **Yes:** Jose Manuel Rodriguez-Llanes

**Dear all, it’s our gratitude for all of you for spending your valuable time to our paper. It helps us to make strong. Thank you**

**Stay safe**
