# Supplementary material for: Assessment of undergraduate student knowledge, attitude, and practices towards COVID-19 in Debre Berhan University, Ethiopia
Source: PLoS One. 2021 May 18;16(5):e0250444. doi: 10.1371/journal.pone.0250444 (PMC8130923; doi:10.1371/journal.pone.0250444)
Supplement: Attachment — Submitted filename: final review.docx [file pone.0250444.s004.docx]

Dear authors,

You have made a great effort with this new article submission. You have included in your submission a marked-up copy of your manuscript highlighting changes and a rebuttal letter responding also to each point raised by the editor and reviewers. Both documents show clearly your new work based in all editor and reviewers’ previous observations. You have improved especially theoretical background and methodological aspects and analysis following our suggestions.

The information regarding written informed consent for your participants and the explanation regarding ethical issues is now clear. The questionnaire in English is so helpful to understand more in depth the results. Following the suggestion of the editor and reviewers you have made extensive edition by a native speaker.

The abstract has been improved following the reviewers’ suggestions. Authors have included the main objective of the article and now there is information regarding findings also in the abstract.

The theoretical background has now new references giving the paper more consistency and comparativeness with other regional areas. Now you have used the latest report of data including different regions and you have modified the WHO strategies.

In the Methods section there is new information answering many diverse comments from the reviewers. You have improved this section correctly, especially regarding participant recruitment method and demographic details. Tables about participants and the recruitment help in this way. The explanation on sample size and power calculation is also clear now. Information regarding how the variables were coded is now improved and the clarification about the multivariable regression instead of multivariate analysis is now clear. The information about the questionnaire is now improved and uploaded in the system. The discussion has now more in-depth analysis, including different finding around the world linked with your results.

The conclusion and recommendation section needs more development. You have improved the conclusions, but it is to short and the recommendations are still so vague. You could start this section confirming (or not) if your main objective (included now in the abstract) has been reached with your research work. Later you could also comment if you have found some correlations or not in your work. Finally, you could provide more details in your recommendations.
