# Supplementary material for: Assessment of undergraduate student knowledge, attitude, and practices towards COVID-19 in Debre Berhan University, Ethiopia
Source: PLoS One. 2021 May 18;16(5):e0250444. doi: 10.1371/journal.pone.0250444 (PMC8130923; doi:10.1371/journal.pone.0250444)
Supplement: Attachment — Submitted filename: Grammar and Conclusion.docx [file pone.0250444.s005.docx]

(Studies)	A study conducted in different parts of the world revealed that Knowledge regarding COVID-19 was adequate among most students and the vast majority of the participants also held an optimistic attitude towards the COVID-19 [

Socio-demographics factor like age, residence, sex, marital status, educational level, field of study, income, family size, religion

The social media should also work by giving a timely and reliable data to the student .A large population based study needs to be conducted to assess the community KAP level at l
