# Supplementary material for: Assessment of undergraduate student knowledge, attitude, and practices towards COVID-19 in Debre Berhan University, Ethiopia
Source: PLoS One. 2021 May 18;16(5):e0250444. doi: 10.1371/journal.pone.0250444 (PMC8130923; doi:10.1371/journal.pone.0250444)
Supplement: Attachment — Submitted filename: Response to Reviewers2.docx [file pone.0250444.s006.docx]

**Dear Academic Editor!**

**Plose one**

**Response to Academic Editor**

I am pleased to resubmit(R2) for publication version of “**Assessment of undergraduate student knowledge, practices, and attitude towards COVID-19 in Debre Berhan University**, Ethiopia for a review as original research in **Plose one**

.The comments of the editor and reviewer were highly insightful and enabled us to greatly improve the quality of our manuscript. Therefore, based on the editor and reviewer concerns we have made extensive edition in our manuscript. The comments of the editor and reviewer r were highly insightful and enabled us to greatly improve the quality of our manuscript. Therefore, based on the editor and reviewer concerns we have made extensive edition in our manuscript. We have edited using the free editing chance that is given to researcher until august 31 at Research Square is currently offering free use of an artificial intelligence editing tool during the Beta period called Automated Editing tool using the following links <https://www.researchsquare.com/static/pdf/automated-editing-tool-instructions.pdf> . Additionally, we have extensively edited the manuscript by a professional language editor, at **Excision Editing** (a fluent, native Australian, English-language speaker thoroughly edited the manuscript for language usage, spelling, and grammar) before submitting the revised version. The formatting of the text and document (text sizes and grammatical errors) were also edited. His name is **called Dr. Ryan Bell(CEO and Chief Editor Excision Editing)**

In the following pages, we have addressed yours’ concerns in a point by point format.

We look forward to hearing from you at your earliest convenience.

Thank you for your consideration of this manuscript!

Kind regards,

**Yared Asmare Aynalem.**

On behalf of authors

**Editor’s comment**

ONE-D-20-14158R1
Assessment of undergraduate student knowledge, practices, and attitude towards COVID-19 in Debre Berhan University, Ethiopia
PLOS ONE

Dear Dr. Aynalem,

Thank you for submitting your manuscript to PLOS ONE. After careful consideration, we feel that it has merit but does not fully meet PLOS ONE’s publication criteria as it currently stands. Therefore, we invite you to submit a revised version of the manuscript that addresses the points raised during the review process.

Response: Thank you very much for allowing us to revise our manuscript again. We have tried to response each concerns as much as possible. We have submitted a revised version of the manuscript.

The reviewers have very much appreciated the effort the authors have made in considering and introducing the suggestion proposed- however, they still find the language unclear and in need of another grammatical revision. I understand that a professional proofreader reviewed the paper, but several sentences across the paper remain below an acceptable level of clarity. One of the reviewers identified so many unclear sentences and phrases that felt frustrated, and this may be something the readers may feel as well.

Response: Thank you very much. We have edited it again using the free editing chance that is given to researcher until august 31 at Research Square is currently offering free use of an artificial intelligence editing tool during the Beta period called Automated Editing tool using the following links <https://www.researchsquare.com/static/pdf/automated-editing-tool-instructions.pdf> .

Additionally, please note the comments by the reviewers about the new information introduced regarding the sampling strategy, and I would urge you to examine the responses offered previously about the justification for such sampling strategy, as I felt such response was not as detailed and robust as ideal. Finally, consider the suggestion by the reviewers to evaluate the Discussion and recommendations, as they remain short in length and unconnected to the aims and results of the paper, as well as existing literature.

Response: thanks our respected editor .We the authors tried to address all the concerns as much as we ca. please see the track change

As PLOS ONE focuses on scientific quality rather than novelty or impact, consideration towards the clarity, thoroughness and addition to the existing body of evidence in the topic of the paper become even more fundamental. Please submit your revised manuscript by Oct 11 2020 11:59PM. If you will need more time than this to complete your revisions, please reply to this message or contact the journal office at [plosone@plos.org](mailto:plosone@plos.org). When you're ready to submit your revision, log on to <https://www.editorialmanager.com/pone/> and select the 'Submissions Needing Revision' folder to locate your manuscript file.

Please include the following items when submitting your revised manuscript:

- A rebuttal letter that responds to each point raised by the academic editor and reviewer(s). You should upload this letter as a separate file labeled 'Response to Reviewers'.
- A marked-up copy of your manuscript that highlights changes made to the original version. You should upload this as a separate file labeled 'Revised Manuscript with Track Changes'.
- An unmarked version of your revised paper without tracked changes. You should upload this as a separate file labeled 'Manuscript'.
- Response: Thank you. We have included ‘Response to Reviewers', 'Revised Manuscript with Track Changes ad Manuscript separately

If you would like to make changes to your financial disclosure, please include your updated statement in your cover letter. Guidelines for resubmitting your figure files are available below the reviewer comments at the end of this letter.

If applicable, we recommend that you deposit your laboratory protocols in [protocols.io](http://protocols.io/) to enhance the reproducibility of your results. Protocols.io assigns your protocol its own identifier (DOI) so that it can be cited independently in the future. For instructions see: <http://journals.plos.org/plosone/s/submission-guidelines#loc-laboratory-protocols>

We look forward to receiving your revised manuscript.

Kind regards,

Enrique Castro-Sánchez
Academic Editor
PLOS ONE


[Note: HTML markup is below. Please do not edit.]

Reviewers' comments:

Reviewer's Responses to Questions

**Comments to the Author**

1. If the authors have adequately addressed your comments raised in a previous round of review and you feel that this manuscript is now acceptable for publication, you may indicate that here to bypass the “Comments to the Author” section, enter your conflict of interest statement in the “Confidential to Editor” section, and submit your "Accept" recommendation.

Reviewer #1: (No Response)

Reviewer #2: All comments have been addressed

Reviewer #3: All comments have been addressed

Reviewer #4: All comments have been addressed

Reviewer #5: (No Response)

Response: thank you for acknowledging that

2. Is the manuscript technically sound, and do the data support the conclusions?

The manuscript must describe a technically sound piece of scientific research with data that supports the conclusions. Experiments must have been conducted rigorously, with appropriate controls, replication, and sample sizes. The conclusions must be drawn appropriately based on the data presented.

Reviewer #1: Yes

Reviewer #2: Yes

Reviewer #3: Yes

Reviewer #4: Yes

Reviewer #5: Partly

Response: thank you for acknowledging that .we the authors tried to address it again

3. Has the statistical analysis been performed appropriately and rigorously?

Reviewer #1: Yes

Reviewer #2: Yes

Reviewer #3: Yes

Reviewer #4: Yes

Reviewer #5: No

Response: thank you for acknowledging that .we the authors tried to address it again

4. Have the authors made all data underlying the findings in their manuscript fully available?

The [PLOS Data policy](http://www.plosone.org/static/policies.action#sharing) requires authors to make all data underlying the findings described in their manuscript fully available without restriction, with rare exception (please refer to the Data Availability Statement in the manuscript PDF file). The data should be provided as part of the manuscript or its supporting information, or deposited to a public repository. For example, in addition to summary statistics, the data points behind means, medians and variance measures should be available. If there are restrictions on publicly sharing data—e.g. participant privacy or use of data from a third party—those must be specified.

Reviewer #1: Yes

Reviewer #2: Yes

Reviewer #3: Yes

Reviewer #4: Yes

Reviewer #5: No

Response: thank you for acknowledging that .we the authors tried to address included all the data

5. Is the manuscript presented in an intelligible fashion and written in standard English?

PLOS ONE does not copyedit accepted manuscripts, so the language in submitted articles must be clear, correct, and unambiguous. Any typographical or grammatical errors should be corrected at revision, so please note any specific errors here.

Reviewer #1: Yes

Reviewer #2: Yes

Reviewer #3: Yes

Reviewer #4: Yes

Reviewer #5: No

Response: Thank you very much. We have edited it again using the free editing chance that is given to researcher until august 31 at Research Square is currently offering free use of an artificial intelligence editing tool during the Beta period called Automated Editing tool using the following links <https://www.researchsquare.com/static/pdf/automated-editing-tool-instructions.pdf>

6. Review Comments to the Author

Please use the space provided to explain your answers to the questions above. You may also include additional comments for the author, including concerns about dual publication, research ethics, or publication ethics. (Please upload your review as an attachment if it exceeds 20,000 characters)

Response: thanks .We tried to address each and every question/concern step by step

Reviewer #1: Thank you much improved
I can see you have put a lot of work in and i think it is useful now.
Response: thank you for acknowledging that dear our respected reviewer

Still riddled with grammar and language issues.
I started pointing out and then got frustrated so below list is not complete. Needs proofreading and correctig again please.

Thank you very much. We have edited it again using the free editing chance that is given to researcher until august 31 at Research Square is currently offering free use of an artificial intelligence editing tool during the Beta period called Automated Editing tool using the following links <https://www.researchsquare.com/static/pdf/automated-editing-tool-instructions.pdf.we> have attached as a supplementary fields the edited

Methods
sample size first paragraph:
Since there is no any study in our countries - correct

Spelling collage - college

Since there is no any study in our countries

Thank you very much. We have corrected all
Reviewer #2: The grammatical errors have been largely addressed. In the recommendation section, the authors should improve to include other dissemination strategies that can be more effective in rural context such as the study sight. See attachment

Response: we the authors would like to acknowledge for your effort to improve our paper. We have got both the attached documents ad we tried to address per your suggestion. We thanks

Reviewer #3: Dear authors,

You have made a great effort with this new article submission. You have included in your submission a marked-up copy of your manuscript highlighting changes and a rebuttal letter responding also to each point raised by the editor and reviewers. Both documents show clearly your new work based in all editor and reviewers’ previous observations. You have improved especially theoretical background and methodological aspects and analysis following our suggestions. The information regarding written informed consent for your participants and the explanation regarding ethical issues is now clear. The questionnaire in English is so helpful to understand more in depth the results. Following the suggestion of the editor and reviewers you have made extensive edition by a native speaker. The abstract has been improved following the reviewers’ suggestions. Authors have included the main objective of the article and now there is information regarding findings also in the abstract. The theoretical background has now new references giving the paper more consistency and comparativeness with other regional areas. Now you have used the latest report of data including different regions and you have modified the WHO strategies. In the Methods section there is new information answering many diverse comments from the reviewers. You have improved this section correctly, especially regarding participant recruitment method and demographic details. Tables about participants and the recruitment help in this way. The explanation on sample size and power calculation is also clear now. Information regarding how the variables were coded is now improved and the clarification about the multivariable regression instead of multivariate analysis is now clear. The information about the questionnaire is now improved and uploaded in the system. The discussion has now more in-depth analysis, including different finding around the world linked with your results.
Response: thank you for acknowledging that dear our respected reviewer. We hope we have tried to address the rest concerns as much as we can
The conclusion and recommendation section needs more development. You have improved the conclusions, but it is to short and the recommendations are still so vague. You could start this section confirming (or not) if your main objective (included now in the abstract) has been reached with your research work. Later you could also comment if you have found some correlations or not in your work. Finally, you could provide more details in your recommendations**.**

Response: thank you for dear our respected reviewer. We hope we have tried to address the rest concerns as much as we can.

Reviewer #4: The authors have addressed my critiques regarding the presentation and analysis of the data. The revised presentation of the sample, variables and the analysis is clear and apropriate.

Response: thank you for acknowledging that dear our respected reviewer. We hope we have tried to address the rest concerns as much as we can

Reviewer #5**:** The KAP Survey Model stands for Knowledge, Attitudes, and Practices. The title may reflect this order. Also it is more logical.

**Response: dear reviewer thanks for your critical comments, we have modified it. See the track change**

The manuscript lacks a more convincing argumentation on why COVID19' KAP is important amongst students. In other words, why to study that population and not another one, or the general population, where poverty is expected to be more prevalent and interventions high priority.
**Response:** dear reviewer we thanks for your feedback. The rational of studying the students KAP is stated as follows: The pandemic also puts the whole educational system in to difficult situations; predominantly, undergraduate students represented a special group that was at the ages to get independence and freedom of life but with inadequate experiences. Consequently, their KAP were suggested to be significantly affected by the pandemic, which needed to be explored. The other thing is that we have assesse the KAP of the community after addressing this group.
In Methods (section entitled ‘Sample size determination and sampling procedure (procurement procedures’)
The authors wrote as new text ‘Since we have used 50% proportion, our sample can be considered as a representative of a larger population’. This sentence makes no sense and the authors should remove it from the manuscript. Setting a 50% level for each of the variables in the survey is helpful as it is a conservative approach when no previous knowledge is available on expected levels of a variable within a population. Setting that level ensures that the sample size will be sufficient for any level as a 50% prevalence or proportion generates the highest requirement for sample, keeping other known parameters constant.

Response: thank you dear our respected reviewer. We have removed it per your golden feedback

Importantly, the authors mentioned several times (in manuscript and reply to reviewers) that they intended to represent the student population in that university. If that is so, then the described two stage sampling strategy selected by the authors is not self-weighted, and the authors need to calculate weights and incorporate those into their analysis. In addition there is attrition from the 'planned' sample to the 'collected' sample but little detail on circumstances (not present in dormitory at time of interview, refused to participate, provided incomplete answers or partial answers).

Response: Dear our kid reviewer thank you for your contractive concern .as you describe the Our study sample was obtained by a two-stage cluster sampling technique In the first stage, out of ten collages, four of them (collage of engendering, computing science, business and economics and collage of agriculture) were selected by simple random sampling, and the sample size was allocated proportionally to obtain the required sample size from each selected college. The sampling fraction is approximately equal to seven for all colleges. We also add 1.5 **design effects.** In the second stage, the study participants were selected from each year (see supplmetry file 1) and sections of the selected collage using a systematic random sampling technique every 7^th^ student from each college registrar office log-book. After that, a dorm-to-dorm visit was used to obtain the sampled students. Students in each selected collage that were present during the data collection period and were able to give responses were included in the study. Those who had mental or physical disabilities and could not fill out the questionnaires (unwilling to participate in to the study) were excluded from the study. Frankly speaking we have proportionally allocated the sample size for each collage. But ,we didn’t code for each it like that .sorry please understand us dear reviewer

In the Discussion section, the authors wrote the following new text:
‘A supportive results were reported in China [13],Jordan [15] and India [12]. However, finding from Saudi Arabia 31.9%[16], Middle Eastern Arabic countries 17.0%[17],India 19.36% [18],in Tanzania [19] and in Uganda [20] showed that most of the community had inadequate Knowledge .This might be due to the late confirmed case report of COVID-19 in Ethiopia which might gave a time to know about the disease. Additionally, the devastating news reported about the disease, and the WHO declaration of the disease as a pandemic due to its sever pathogenicity and communicability [7, 8]. might also have increased the students’ knowledge.’ The first sentence above is not good English but the authors claimed the paper to be edited and polished by professionals. It does not seem to be the case here. Another example is below.‘This finding is in line with a study done in Indonesian[31], Nepalese[32], Sudan[30] and South Wollo Zone[33]’. Overall, the discussion is gaining some importance and becoming more balanced but requires better expression of the main ideas, and improving clarity. I see many punctuation errors.

Response: thank you very much .The comments of the reviewer were highly insightful and enabled us to greatly improve the quality of our manuscript. Thank you for that .Therefore, based on the reviewer concerns we have made extensive edition in our manuscript. We have edited using the free editing chance that is given to researcher until august 31 at Research Square is currently offering free use of an artificial intelligence editing tool during the Beta period called Automated Editing tool using the following links <https://www.researchsquare.com/static/pdf/automated-editing-tool-instructions.pdf> . We have attached it as a supplementary file. See it

References no. 1, 10 and 11, seem incomplete.

Response: Dear our respected reviewers, we would like to thanks once again for your critical review of the paper. We have cited with the full citation (we have removed the [www.way](http://www.way) of citation & we cited it again. See it

7. PLOS authors have the option to publish the peer review history of their article ([what does this mean?](https://journals.plos.org/plosone/s/editorial-and-peer-review-process#loc-peer-review-history)). If published, this will include your full peer review and any attached files.


If you choose “no”, your identity will remain anonymous but your review may still be made public.


**Do you want your identity to be public for this peer review?** For information about this choice, including consent withdrawal, please see our [Privacy Policy](https://www.plos.org/privacy-policy).

Reviewer #1: **Yes:** PW Hodkinson

Reviewer #2: **Yes:** Dr John Bosco Azigwe

Reviewer #3: **Yes:** Ramon Flecha

Reviewer #4: No

Reviewer #5: **Yes:** Jose Manuel Rodriguez-Llanes

Response: Dear our respected reviewers, we would like to thanks for your effort of giving the constructive feedback throughout the paper progresses. Without your feedback the output of this finding may loss its strength. For that thank you

Stay safe

Thanks
