# Supplementary material for: Assessment of undergraduate student knowledge, attitude, and practices towards COVID-19 in Debre Berhan University, Ethiopia
Source: PLoS One. 2021 May 18;16(5):e0250444. doi: 10.1371/journal.pone.0250444 (PMC8130923; doi:10.1371/journal.pone.0250444)
Supplement: Attachment — Submitted filename: Response to reviewers (R3).docx [file pone.0250444.s007.docx]

**Dear Academic Editor!**

**Plose one**

**Response to Academic Editor**

I am pleased to resubmit(R3) for publication version of “**Assessment of undergraduate student knowledge, practices, and attitude towards COVID-19 in Debre Berhan University**, Ethiopia for a review as original research in **Plose one**

The comments of the editor and the reviewers were highly insightful and enabled us to greatly improve the quality of our manuscript. Therefore, based on the editor’s and the reviewers’ concerns we have made extensive edition in our manuscript. The comments of the editor and the reviewers were highly insightful and enabled us to greatly improve the quality of our manuscript. Therefore, based on the editor’s and the reviewers’ concerns we have made extensive edition in our manuscript. Especially we have extensively edited the manuscript again by a professional language editor, at **Excision Editing** (a fluent, native Australian, English-language speaker thoroughly edited the manuscript for language usage, spelling, and grammar) before submitting the revised version. The formatting of the text and document (text sizes and grammatical errors) were also edited. His name is **called Dr. Ryan Bell(CEO and Chief Editor Excision Editing)**

In the following pages, we have addressed yours’ concerns in a point by point format.

We look forward to hearing from you at your earliest convenience.

Thank you for your consideration of this manuscript!

Kind regards,

**Yared Asmare Aynalem.**

On behalf of authors

PONE-D-20-14158R2
Assessment of undergraduate student knowledge, attitude, and practices, towards COVID-19 in Debre Berhan University, Ethiopia
PLOS ONE

Dear Dr. Aynalem,

Thank you for submitting your manuscript to PLOS ONE. After careful consideration, we feel that it has merit but does not fully meet PLOS ONE’s publication criteria as it currently stands. Therefore, we invite you to submit a revised version of the manuscript that addresses the points raised during the review process.

Response: Thank you very much for allowing us to revise our manuscript again. We have tried to response each concerns as much as possible. We have submitted a revised version of the manuscript.

The reviewers have emphasised once more 3 different areas where the manuscript does require improvement and clarifications:

- Presentation and overall writing quality of the paper, with references still incomplete, punctuation errors etc - please assume the paper is going to be read widely, and by many different readers which may include policymakers, government officials, other colleagues and patients/citizens, so I would encourage care on the overall appearance.

Response: Thank you very much. We the authors tried to edit the whole manuscript per the reviewer and the editor suggestion

- Further details so that readers understand how the authors made their design selfweighted. Bit more clarity is needed. The argumentation for a 50% proportion is not obvious for a non expert reader and should be better supported with an explanatory reference.

Respose Dear our respected review ereditor , thanks for spending your time to this extent. Which helps to improve our paper as your concern, we the authors tried to make the reference as completed as possible. Additionally, we have made an extensive edition of the paper including the punctuation issue. We also support the argumentation of using a 50% proportion with citation. For more please also see the color change

- Review again the Discussion and recommendations section, because they remain short in length and not directly connected with the main objective included in the abstract.

Response: Thank you very much. We have rewrite the Discussion and recommendations section as detailed ad possible

Please submit your revised manuscript by Nov 14 2020 11:59PM. If you will need more time than this to complete your revisions, please reply to this message or contact the journal office at [plosone@plos.org](mailto:plosone@plos.org). When you're ready to submit your revision, log on to <https://www.editorialmanager.com/pone/> and select the 'Submissions Needing Revision' folder to locate your manuscript file.

Please include the following items when submitting your revised manuscript:

- A rebuttal letter that responds to each point raised by the academic editor and reviewer(s). You should upload this letter as a separate file labeled 'Response to Reviewers'.
- A marked-up copy of your manuscript that highlights changes made to the original version. You should upload this as a separate file labeled 'Revised Manuscript with Track Changes'.
- An unmarked version of your revised paper without tracked changes. You should upload this as a separate file labeled 'Manuscript'.

Response: Thank you. We have included ‘Response to Reviewers', 'Revised Manuscript with Track Changes & Manuscript separately

If you would like to make changes to your financial disclosure, please include your updated statement in your cover letter. Guidelines for resubmitting your figure files are available below the reviewer comments at the end of this letter.
Response: Thank you for the concern. There is no any change
If applicable, we recommend that you deposit your laboratory protocols in [protocols.io](http://protocols.io/) to enhance the reproducibility of your results. Protocols.io assigns your protocol its own identifier (DOI) so that it can be cited independently in the future. For instructions see: <http://journals.plos.org/plosone/s/submission-guidelines#loc-laboratory-protocols>

We look forward to receiving your revised manuscript.

Kind regards,

Enrique Castro-Sánchez
Academic Editor

Response: Thank you for the concern. Not applicable
PLOS ONE

Journal Requirements:

1) We note that Table 2 appears to be mislabeled as a second Table 1. Please revise the table name for clarity.


Response: Thank you. We have labeled it. See the color change
Reviewers' comments:

Reviewer's Responses to Questions

**Comments to the Author**

1. If the authors have adequately addressed your comments raised in a previous round of review and you feel that this manuscript is now acceptable for publication, you may indicate that here to bypass the “Comments to the Author” section, enter your conflict of interest statement in the “Confidential to Editor” section, and submit your "Accept" recommendation.

Reviewer #2: All comments have been addressed

Reviewer #3: (No Response)

Reviewer #5: (No Response)

Response: thank you for acknowledging that

2. Is the manuscript technically sound, and do the data support the conclusions?

The manuscript must describe a technically sound piece of scientific research with data that supports the conclusions. Experiments must have been conducted rigorously, with appropriate controls, replication, and sample sizes. The conclusions must be drawn appropriately based on the data presented.

Reviewer #2: Yes

Reviewer #3: Yes

Reviewer #5: Partly

Response: thank you for acknowledging that .we the authors tried to address it again

3. Has the statistical analysis been performed appropriately and rigorously?

Reviewer #2: Yes

Reviewer #3: Yes

Reviewer #5: I Don't Know

Response: thank you for acknowledging that .

4. Have the authors made all data underlying the findings in their manuscript fully available?

The [PLOS Data policy](http://www.plosone.org/static/policies.action#sharing) requires authors to make all data underlying the findings described in their manuscript fully available without restriction, with rare exception (please refer to the Data Availability Statement in the manuscript PDF file). The data should be provided as part of the manuscript or its supporting information, or deposited to a public repository. For example, in addition to summary statistics, the data points behind means, medians and variance measures should be available. If there are restrictions on publicly sharing data—e.g. participant privacy or use of data from a third party—those must be specified.

Reviewer #2: Yes

Reviewer #3: Yes

Reviewer #5: No

Response: thank you for acknowledging that .we the authors tried to address included all the data

5. Is the manuscript presented in an intelligible fashion and written in standard English?

PLOS ONE does not copyedit accepted manuscripts, so the language in submitted articles must be clear, correct, and unambiguous. Any typographical or grammatical errors should be corrected at revision, so please note any specific errors here.

Reviewer #2: Yes

Reviewer #3: Yes

Reviewer #5: Yes

Response: thank you for acknowledging that

6. Review Comments to the Author

Please use the space provided to explain your answers to the questions above. You may also include additional comments for the author, including concerns about dual publication, research ethics, or publication ethics. (Please upload your review as an attachment if it exceeds 20,000 characters)

Response: thank you .We tried to answer the reviewers concern step by step

Reviewer #2: The authors in their recommendations should be more specific on community based strategies for information dissemination on covid19

Response: dear editor very thank you for your golden feedback. We the authors tried to make it as specific as possible. Please see the color change

Reviewer #3: Dear authors,
I have seen the new version of your article (R2) with track changes and without it. You have made extensive edition in your manuscript using the free use of an artificial intelligence editing tool. The new extensive edited manuscript by a professional language editor is so valuable. You have worked hard in the final proposal in this third stage. I suggest to review again the Discussion and recommendations section, because they remain short in lenght and not directly connected with your main objective included in the abstract.

Response: thank you for acknowledging that .we the authors tried to address the issue .we reviewed again the Discussion and recommendations section, to make it as long as possible and to make it connected with our main objective included in the abstract. see the color.

Reviewer #5: Thanks for the opportunity to revise this MS.
The references are yet incomplete. I can still see punctuation errors in the MS and the authors need to provide further details so that readers understand how they made their design selfweighted. Bit more clarity is needed. The argumentation for a 50% proportion is not obvious for a non expert reader and should be better supported with a explanatory reference.

Response: Dear our respected reviewer, we are glad to have a reviewer like you .thanks for spending your time to this extent. Which helps to improve our paper.as your concern, we the authors tried to make the reference as completed as possible. additionally, we have made an extensive edition of the paper including the punctuation issue. We also support the argumentation of using a 50% proportion with citation. For more please also see the color change

7. PLOS authors have the option to publish the peer review history of their article ([what does this mean?](https://journals.plos.org/plosone/s/editorial-and-peer-review-process#loc-peer-review-history)). If published, this will include your full peer review and any attached files.


If you choose “no”, your identity will remain anonymous but your review may still be made public.

Response: thank you .Yes


**Do you want your identity to be public for this peer review?** For information about this choice, including consent withdrawal, please see our [Privacy Policy](https://www.plos.org/privacy-policy).

Reviewer #2: **Yes:** Dr John Bosco Azigwe

Reviewer #3: **Yes:** Ramon Flecha

Reviewer #5: **Yes:** Jose Manuel Rodriguez-Llanes

Response: thank you for your volunteer of your **identity to be public for this peer review**
