# Supplementary material for: Assessment of undergraduate student knowledge, attitude, and practices towards COVID-19 in Debre Berhan University, Ethiopia
Source: PLoS One. 2021 May 18;16(5):e0250444. doi: 10.1371/journal.pone.0250444 (PMC8130923; doi:10.1371/journal.pone.0250444)
Supplement: Attachment — Submitted filename: Response to reviewers (R4).docx [file pone.0250444.s008.docx]

**Dear Academic Editor!**

**PLOSE ONE**

**Response to Academic Editor**

I am pleased to resubmit revision (R4) for publication version of “**Assessment of undergraduate student knowledge, practices, and attitude towards COVID-19 in Debre Berhan University**, Ethiopia for a review as original research in **Plose one .**The comments of the editor were highly insightful and enabled us to greatly improve the quality of our manuscript. Therefore, based on the editor’s concerns we have made extensive edition in our manuscript. Especially we have extensively edited the manuscript again for language usage, spelling, and grammar before submitting the revised version. The formatting of the text and document (text sizes and grammatical errors) were also edited. In the following pages, we have addressed yours’ concerns in a point by point format.

We look forward to hearing from you at your earliest convenience.

Thank you for your consideration of this manuscript!

Kind regards,

**Yared Asmare Aynalem.**

On behalf of authors

PONE-D-20-14158R3

Assessment of undergraduate student knowledge, attitude, and practices, towards COVID-19 in Debre Berhan University, Ethiopia

PLOS ONE

Dear Dr. Aynalem,

Thank you for submitting your manuscript to PLOS ONE. After careful consideration, we feel that it has merit but does not fully meet PLOS ONE’s publication criteria as it currently stands. Therefore, we invite you to submit a revised version of the manuscript that addresses the points raised during the review process.

Response: Thank you very much for allowing us to revise our manuscript again. We have tried to response each concerns as much as possible. We have submitted a revised version of the manuscript

I thank you for the revisions that you have conducted, but I would urge you once more to carefully review the manuscript in terms of clarity and grammar. This is not the usual request for authors to ensure that the English language in the manuscript is adequate, but to ensure that the manuscript is readable and understandable. The paper has had several rounds of reviews and opportunities for these issues to be resolved, so I would be grateful if you could ensure they are resolved. It is disconcerting to find so many issues despite assurances that they have been resolved.

Response: Thank you for allowing us to edit it again. The comments of the editor and the reviewers were highly insightful and enabled us to greatly improve the quality of our manuscript. Therefore, based on your concerns we have made extensive edition in our manuscript. Your comments were highly insightful and enabled us to greatly improve the quality of our manuscript. Therefore, based on the editor’s concerns we have made extensive edition in our manuscript. Especially we have extensively edited the manuscript again for language clarity thoroughly edited the manuscript for language usage, spelling, and grammar before submitting the revised version. The formatting of the text and document (text sizes and grammatical errors) were also edited.

In addition to the comments and recommendations mentioned by the reviewers, I have read the manuscript again and I would like the authors to resolve these pending matters:

Response: Dear our respected editor thank you very much for raising the following issues

Background

2nd para, why is the mortality compared with the flu?

Response: Dear our respected editor thanks you very much. The reason for comparing COVID-19 with the flu is that to show the proportion of mortality as a new outbreak. At the time of seasonal flue outbreak the mortality rate were almost 1%.more over covid mortality rate is almost more than 7 percent .This death rate is particularly alarming. If you are still not comfortable with this sentence, we will remove it.

End of 2nd para, and start of 3rd para, the data does not match- please review and clarify.

Response: Thanks you very much our respected editor .We have rewrite it again; please see the color change from the main document

Ethics statement

Review wording- "ethical approval commute" should be committee - please review across the manuscript. Review the paragraph for clarity.

Response: Thanks you very much our respected editor. We have copy edited it. see also the color change from the ma document

Response: Thank you dear editor. We have clearly descried the contribution on the on the online author contribution form.

**Background**

2nd para, says "2215 countries", must be a typo.

Response: thanks our beloved editor We have rewritten it. Please See the color change

Reference in pg 16 [9,10] appears in cursive font, please edit.

Response: Thanks .We has corrected it. Please see the color change

End of page: "study conducted in different parts of their world" - say "international" or say which parts of the world.

Response: Thanks .We tried to rephrase & replace with your suggestive appropriate word so called "international"

Methods

Please remove "Abve sea level and temp" unless relevant to the study. Please edit "Collage of ..." To "College of..."

**Response: Thanks .We has corrected it. See the color change**

Justify socio-demographic variables, marital status, religion - why collected?

Response: we would like to thanks our editor for raising these important concerns. The rational for collecting socio-demographic variables, marital status, and religion is as follows

1. In our country context, the distribution of COVID_19 is high among aged individual & most of adult generation perceived that they are less risky of contracting with it. For that they didn’t want to get even information on it. Therefore, we have collected it as a factor.
2. Most of the community including students talk as COVID is a sin of us.it is from alah,God. So that, not practicing a percussion. This document and risk assessment tool provides practical guidance and recommendations to support the special role of religious leaders, faith-based organizations, and faith communities in COVID-19 education, preparedness, and response.
3. Additionally, differ study conducted globally, shows that socio-demographic variables, marital status, religion are linked with COVID-19 KAP. Here, we examine how KAP varies across fundamental socio-demographic characteristics, including age, sex, civil status, individual disposable income, religion of. We have collected it to for identifying the gap & to intervene on it .

Was the data collection validated?

Was tool validated in students or only faculty?

"Ensure genuine replies..." - how did they do that?

Thanks dear reviewer..!!! The proposed tool was developed and validated by a multidisciplinary working group of infectious disease physicians, lecturers (Public health, nurse & environmental health professionals, and infectious diseases Public health professionals. The working group regularly reviewed literature to select important characteristics and outcomes for inclusion. We also used a

The development phase was conducted at Debre birhan University health science collage and the pilot validation phase was conducted at the nursing department students. We have also done a pretest from 5 % of the student. The team members formed a working group that met via telephone or video conferences at least biweekly

Ethics consideration

Commute = committee,Paper says participation anonymous but later on it mentioned that collects personal details like phone? Please clarify

Response: Thanks .We has corrected it. See the color change

Results

What was the total number of participants approached, to give the 86.1% rate? 634

Response: Thanks dear editor.as we have stated in sample size determination, we have included approached to a total of 634 participate.

Discussion

End of 1st para: please add evidence that "Students get regular update through the medias"

Response: Dear editor thanks for raising the concern. We have given a possible justifications based o our experience of giving the student about the outbreak COVID-19. We have given to them a health education program for all faculty of the university before the school was closed. Possibly this may increase their knowledge

2nd and 3rd para need punctuation mark at the end.

Response : Dear editor thanks for raising the concern. We have tried to edit all parts of the paper.

Response: thank you for your critical review dear editor. We have corrected it .See also the color change

Please submit your revised manuscript by Jan 24 2021 11:59PM. If you will need more time than this to complete your revisions, please reply to this message or contact the journal office at plosone@plos.org. When you're ready to submit your revision, log on to https://www.editorialmanager.com/pone/ and select the 'Submissions Needing Revision' folder to locate your manuscript file.

Please include the following items when submitting your revised manuscript:

A rebuttal letter that responds to each point raised by the academic editor and reviewer(s). You should upload this letter as a separate file labeled 'Response to Reviewers'.

A marked-up copy of your manuscript that highlights changes made to the original version. You should upload this as a separate file labeled 'Revised Manuscript with Track Changes'.

An unmarked version of your revised paper without tracked changes. You should upload this as a separate file labeled 'Manuscript'.

Response: Thank you. We have included ‘Response to Reviewers', 'Revised Manuscript with Track Changes & Manuscript separately

If you would like to make changes to your financial disclosure, please include your updated statement in your cover letter. Guidelines for resubmitting your figure files are available below the reviewer comments at the Editorial manager (<https://www.editorialmanager.com/pone/>)

Response: Thank you. We dot like to change the financial disclosure

We look forward to receiving your revised manuscript.

Kind regards,

Enrique Castro-Sánchez

Academic Editor

PLOS ONE

**Reviewers' comments:**

**Reviewer's Responses to Questions**

**Comments to the Author**

1. If the authors have adequately addressed your comments raised in a previous round of review and you feel that this manuscript is now acceptable for publication, you may indicate that here to bypass the “Comments to the Author” section, enter your conflict of interest statement in the “Confidential to Editor” section, and submit your "Accept" recommendation.

Reviewer #3: All comments have been addressed

Response: dear reviewer, we thank you for spending your valuable time to improve this paper. We have a grate appreciation for helping us

2. Is the manuscript technically sound, and do the data support the conclusions?

The manuscript must describe a technically sound piece of scientific research with data that supports the conclusions. Experiments must have been conducted rigorously, with appropriate controls, replication, and sample sizes. The conclusions must be drawn appropriately based on the data presented.

Reviewer #3: Yes

Response: Thank you dear reviewer

3. Has the statistical analysis been performed appropriately and rigorously?

Reviewer #3: Yes

Response: Thank you dear reviewer

4. Have the authors made all data underlying the findings in their manuscript fully available?

The PLOS Data policy requires authors to make all data underlying the findings described in their manuscript fully available without restriction, with rare exception (please refer to the Data Availability Statement in the manuscript PDF file). The data should be provided as part of the manuscript or its supporting information, or deposited to a public repository. For example, in addition to summary statistics, the data points behind means, medians and variance measures should be available. If there are restrictions on publicly sharing data—e.g. participant privacy or use of data from a third party—those must be specified.

Reviewer #3: Yes

Response: Thank you dear reviewer

5. Is the manuscript presented in an intelligible fashion and written in standard English?

PLOS ONE does not copyedit accepted manuscripts, so the language in submitted articles must be clear, correct, and unambiguous. Any typographical or grammatical errors should be corrected at revision, so please note any specific errors here.

Reviewer #3: Yes

Response: Thank you dear reviewer

6. Review Comments to the Author

Please use the space provided to explain your answers to the questions above. You may also include additional comments for the author, including concerns about dual publication, research ethics, or publication ethics. (Please upload your review as an attachment if it exceeds 20,000 characters)

Reviewer #3: Authors have adequately addressed my comments in this new version of the article. Discussion section provides more concrete information.

Response: Thank you dear reviewer. It is your effort too

7. PLOS authors have the option to publish the peer review history of their article (what does this mean?). If published, this will include your full peer review and any attached files.

If you choose “no”, your identity will remain anonymous but your review may still be made public.

Do you want your identity to be public for this peer review? For information about this choice, including consent withdrawal, please see our Privacy Policy.

Reviewer #3: Yes: Ramon Flecha

Response: Thank you dear reviewer

Dear all (both the reviewer ad editors),we would like to appreciate all your effort for your effort to improve our paper .may God paid all your effort. We have get differ research skills from your point of view

Thanks
