# Supplementary material for: Assessment of undergraduate student knowledge, attitude, and practices towards COVID-19 in Debre Berhan University, Ethiopia
Source: PLoS One. 2021 May 18;16(5):e0250444. doi: 10.1371/journal.pone.0250444 (PMC8130923; doi:10.1371/journal.pone.0250444)
Supplement: Attachment — Submitted filename: Response to reviewers (R5).docx [file pone.0250444.s010.docx]

**Dear Academic Editor!**

**Plose one**

**Response to Academic Editor**

I am pleased to resubmit(R5) for publication version of “**Assessment of undergraduate student knowledge, practices, and attitude towards COVID-19 in Debre Berhan University**, Ethiopia for a review as original research in **Plose one**

The comments of the editor were highly insightful and enabled us to greatly improve the quality of our manuscript. Therefore, based on the editor’s concerns we have made extensive edition in our manuscript. The comments of the editor and were highly insightful and enabled us to greatly improve the quality of our manuscript. Therefore, based on the editor’s and the reviewers’ concerns we have made extensive edition in our manuscript. Especially we have extensively edited the manuscript again per editors feedback thoroughly edited the manuscript for language usage, spelling, and grammar) before submitting the revised version. The formatting of the text and document (text sizes and grammatical errors) were also edited. In the following pages, we have addressed yours’ concerns in a point by point format.

We look forward to hearing from you at your earliest convenience.

Thank you for your consideration of this manuscript!

Kind regards,

**Yared Asmare Aynalem.**

On behalf of authors

PONE-D-20-14158R4
Assessment of undergraduate student knowledge, attitude, and practices, towards COVID-19 in Debre Berhan University, Ethiopia
PLOS ONE

Dear Dr. Aynalem,

Thank you for submitting your manuscript to PLOS ONE. After careful consideration, we feel that it has merit but once again, it does not fully meet PLOS ONE’s publication criteria as it currently stands. Therefore, we invite you to submit a revised version of the manuscript that addresses the points raised during the review process.

I have reviewed again, and annotated, the revised version of the manuscript. As you can see, several language and copyediting issues remain, which I have highlighted. The reason why I am emphasizing this aspect of the process is that PLOS ONE manuscripts are published as submitted by the authors, as per the accepted version.

Additionally, some of the responses provided to justify some of the concerns and queries raised are not adequate as they really do not offer a justification.

****************************************************************************************

2nd para, why is the mortality compared with the flu? Response: Dear our respected editor thanks you very much. The reason for comparing COVID-19 with the flu is that to show the proportion of mortality as a new outbreak. At the time of seasonal flue outbreak the mortality rate were almost 1%.more over covid mortality rate is almost more than 7 percent .This death rate is particularly alarming. If you are still not comfortable with this sentence, we will remove it.

**>> Please remove or simply state that the mortality of covid is estimated at more than 7%**

**Response: thanks dear editor. We have modified it. See the track change**

Background

2nd para, says "2215 countries", must be a typo. Response: thanks our beloved editor we have rewritten it. Please see the color change

**>>>The text still says 2215 countries. Please review once more, do you mean 215 countries?**

**Response: our respected editor, a great apologize to the inconvenience**

**It’s to say two hundred fifteen countries**

Justify socio-demographic variables, marital status, religion - why collected?

Response: we would like to thanks our editor for raising these important concerns. The rational for collecting socio-demographic variables, marital status, and religion is as follows

1. In our country context, the distribution of COVID_19 is high among aged individual & most of adult generation perceived that they are less risky of contracting with it. For that they didn’t want to get even information on it. Therefore, we have collected it as a factor.

2. Most of the community including students talk as COVID is a sin of [us.it](http://us.it/) is from alah,God. So that, not practicing a percussion. This document and risk assessment tool provides practical guidance and recommendations to support the special role of religious leaders, faith-based organizations, and faith communities in COVID-19 education, preparedness, and response. **>>>>If that is the case, and there is evidence of students talking about covid19 as a sin, then this argument needs to be included in the paper.**
3. Additionally, differ study conducted globally, shows that socio-demographic variables, marital status, religion are linked with COVID-19 KAP. **>>>>Can you please include references to those studies that indicate that marital status and religion are linked with COVID-19 KAP?**

**Response –dear editor once again we would like to acknowledge for your deep understanding of our paper. Even if we included the above mentioned variables, we dint got a significant association with our regression result. We have described them only them.so that we didn’t cited them. If you consider irrelevant, we may remove the variables .more of the above description that we have put were a rumor with in the community, that is why we want to see them**

**Thank you**

Ethics consideration

Commute = committee, Paper says participation anonymous but later on it mentioned that collects personal details like phone? Please clarify

Response: Thanks .We has corrected it. See the color change

**>>>> There is still a mention to the collection of phone number. The authors did not respond to the query. Why was the phone number of the participants collected?**

**Response : Dear editor thank you for the query. We have use the phone number of the student to trace them .cuse they were isolated to their dormitory**

Results

What was the total number of participants approached, to give the 86.1% rate? 634

Response: Thanks dear [editor.as](http://editor.as/) we have stated in sample size determination, we have included approached to a total of 634 participate.

**>>>The 634 number is not included in the manuscript, can you include?**

**Response: Thank you. We have included it.**

Discussion

End of 1st para: please add evidence that "Students get regular update through the Medias"

Response: Dear editor thanks for raising the concern. We have given a possible justifications based o our experience of giving the student about the outbreak COVID-19. We have given to them a health education program for all faculty of the university before the school was closed. Possibly this may increase their knowledge

**>>>In the Discussion, authors are allowed, and encouraged, to hypothesise about the reasons explaining their findings. However, the authors also need to make sure that the reader knows whether there is any data supporting such explanations. IF you say that "in your experience students get regular update through media", then you should include that in the paper.**

**Response: dear reviewer we tried to cite all justification as per your valuable feedback**


Please submit your revised manuscript by Mar 05 2021 11:59PM. If you will need more time than this to complete your revisions, please reply to this message or contact the journal office at [plosone@plos.org](mailto:plosone@plos.org). When you're ready to submit your revision, log on to <https://www.editorialmanager.com/pone/> and select the 'Submissions Needing Revision' folder to locate your manuscript file.

Please include the following items when submitting your revised manuscript:

- A rebuttal letter that responds to each point raised by the academic editor and reviewer(s). You should upload this letter as a separate file labeled 'Response to Reviewers'.
- A marked-up copy of your manuscript that highlights changes made to the original version. You should upload this as a separate file labeled 'Revised Manuscript with Track Changes'.
- An unmarked version of your revised paper without tracked changes. You should upload this as a separate file labeled 'Manuscript'.

If you would like to make changes to your financial disclosure, please include your updated statement in your cover letter. Guidelines for resubmitting your figure files are available below the reviewer comments at the end of this letter.

If applicable, we recommend that you deposit your laboratory protocols in [protocols.io](http://protocols.io/) to enhance the reproducibility of your results. Protocols.io assigns your protocol its own identifier (DOI) so that it can be cited independently in the future. For instructions see: <http://journals.plos.org/plosone/s/submission-guidelines#loc-laboratory-protocols>

We look forward to receiving your revised manuscript.

Kind regards,

Enrique Castro-Sánchez
Academic Editor
PLOS ONE


Response: Thank you. We have included ‘Response to Reviewers', 'Revised Manuscript with Track Changes & Manuscript separately
