# Supplementary material for: Assessment of undergraduate student knowledge, attitude, and practices towards COVID-19 in Debre Berhan University, Ethiopia
Source: PLoS One. 2021 May 18;16(5):e0250444. doi: 10.1371/journal.pone.0250444 (PMC8130923; doi:10.1371/journal.pone.0250444)
Supplement: Attachment — Submitted filename: Response to reviewers (R8).docx [file pone.0250444.s013.docx]

**Dear Academic Editor!**

**Plose one**

**Response to Academic Editor**

I am pleased to resubmit(R8) for publication version of “**Assessment of undergraduate student knowledge, practices, and attitude towards COVID-19 in Debre Berhan University**, Ethiopia for a review as original research in **Plose one .**The comments of the editor were highly insightful and enabled us to greatly improve the quality of our manuscript. Therefore, based on the editor’s concerns we have made extensive edition in our manuscript. The comments of the editor were highly insightful and enabled us to greatly improve the quality of our manuscript. Therefore, based on the editor’s concerns we have made extensive edition in our manuscript particularly the reference part. The formatting of the text and document (text sizes and grammatical errors) were also edited. In the following pages, we have addressed yours’ concerns in a point by point format.

We look forward to hearing from you at your earliest convenience.

Thank you for your consideration of this manuscript!

Kind regards,

**Yared Asmare Aynalem.**

On behalf of authors

PONE-D-20-14158R5
Assessment of undergraduate student knowledge, attitude, and practices, towards COVID-19 in Debre Berhan University, Ethiopia
PLOS ONE

Dear Dr. Aynalem,

Thank you for submitting your manuscript to PLOS ONE. After careful consideration, we feel that it has merit but does not fully meet PLOS ONE’s publication criteria as it currently stands. Therefore, we invite you to submit a revised version of the manuscript that addresses the points raised during the review process.

Response: Dear our respected editor thanks you very much for considerations. I have submitted a revised version of the manuscript that addresses the points raised during the review process as much as we can.

*The following is an odd sentence and needs to be revised in the discussion: "This might be because of easily accessible to most students at home and everywhere through the mobile internet".*

SPECIFIC ACADEMIC EDITOR COMMENT:

That previous sentence in the discussion that I requested be revised is still not correct. Phrase as "This may be due to ease of access to readily updated information to most students via the internet and social media [34]."

 .

Response: Dear our respected editor thanks you very much for your critical review. We have REVISE that sentence to proper English and make it understandable and resubmit this manuscript as per your suggestion . see the color change

Please include the following items when submitting your revised manuscript:

- A rebuttal letter that responds to each point raised by the academic editor and reviewer(s). You should upload this letter as a separate file labeled 'Response to Reviewers'.
- A marked-up copy of your manuscript that highlights changes made to the original version. You should upload this as a separate file labeled 'Revised Manuscript with Track Changes'.
- An unmarked version of your revised paper without tracked changes. You should upload this as a separate file labeled 'Manuscript'.

 Response: Thank you. We have included ‘Response to Reviewers', 'Revised Manuscript with Track Changes ad Manuscript separately

If you would like to make changes to your financial disclosure, please include your updated statement in your cover letter. Guidelines for resubmitting your figure files are available below the reviewer comments at the end of this letter.

Response: thanks for allowing us to update the financial disclosure. However, there is no any change.
If applicable, we recommend that you deposit your laboratory protocols in [protocols.io](http://protocols.io/) to enhance the reproducibility of your results. Protocols.io assigns your protocol its own identifier (DOI) so that it can be cited independently in the future. For instructions see: <http://journals.plos.org/plosone/s/submission-guidelines#loc-laboratory-protocols>

Response: thank you for recommending depositing the laboratory protocols in protocols.io. But it is not applicable


We look forward to receiving your revised manuscript.

Kind regards,

Frank T. Spradley
Academic Editor

Response: Thank you dear editor .we has set it

PLOS ONE

Journal Requirements:

Please review your reference list to ensure that it is complete and correct. If you have cited papers that have been retracted, please include the rationale for doing so in the manuscript text, or remove these references and replace them with relevant current references. Any changes to the reference list should be mentioned in the rebuttal letter that accompanies your revised manuscript. If you need to cite a retracted article, indicate the article’s retracted status in the References list and also include a citation and full reference for the retraction notice

Response: thank you for the feedback dear our editor.as far as we searched we didn’t cite papers that have been retracted and no any changes to the reference list. We tried to search for papers that might retract from journal. However as far as we searched, we didn’t get it. if any please suggest us & we will remove and replace it .We also tried to make the reference full as the journal guide line. See the track charge

Thank you

Keep safe
